# Outcomes reported in studies of anal high-grade squamous intraepithelial lesions treatments: systematic review

**DOI:** 10.1093/bjsopen/zrag061

**Published:** 2026-07-04

**Authors:** David A Finch, Madhu P Chaudhury, Rebecca Morris, Edward Parkin, Peter Mitchell, Pierre Martin-Hirsch, Andrew G Renehan, Rebecca Fish

**Affiliations:** Division of Cancer Sciences, University of Manchester, Manchester, UK; Department of Colorectal Surgery, Lancashire Teaching Hospitals NHS Foundation Trust, Preston, UK; Division of Population Health, Health Services Research and Primary Care, University of Manchester, Manchester, UK; Division of Population Health, Health Services Research and Primary Care, University of Manchester, Manchester, UK; Division of Population Health, Health Services Research and Primary Care, University of Manchester, Manchester, UK; Department of Gynecological Oncology, Lancashire Teaching Hospitals NHS Foundation Trust, Preston, UK; Division of Cancer Sciences, University of Manchester, Manchester, UK; Colorectal and Peritoneal Oncology Centre (CPOC), The Christie NHS Foundation Trust, Manchester, UK; Colorectal and Peritoneal Oncology Centre (CPOC), The Christie NHS Foundation Trust, Manchester, UK

**Keywords:** core outcome measure, HSIL, anal squamous carcinoma, patient-reported outcome, anal intraepithelial neoplasia, anal neoplasms, outcome studies, outcome measures, Core outcome measure, HSIL, Anal squamous carcinoma, patient-reported outcome

## Abstract

**Introduction:**

Anal high-grade squamous intraepithelial lesion (HSIL) is a precursor of anal squamous cell carcinoma (ASCC). Reported outcomes from treatment trials vary widely, limiting understanding of the effectiveness of different interventions. This systematic review aimed to summarize the outcomes reported in studies evaluating treatments in anal HSIL.

**Methods:**

A systematic review of MEDLINE, Embase, CINAHL, and the Cochrane Library (inception to June 2025) was conducted. Eligible studies evaluated any anal HSIL treatment in any population. Verbatim outcomes and associated definitions, measurement tools, and timepoints were extracted and mapped to standardized outcome terms (SOTs), categorized using The Core Outcome Measures in Effectiveness Trials (COMET) Initiative taxonomy.

**Results:**

Out of 5488 studies screened, 67 studies were included, comprising 40 observational studies, 23 interventional studies (5 randomized controlled trials), and 4 systematic reviews. A total of 966 outcomes were reported, grouped into 170 SOTs. Outcomes were mapped across all five COMET core areas (Death, Physiological/clinical, Life impact, Resource use, and Adverse events), with Physiological/clinical outcomes most frequently represented (131/170 SOTs; 77%). Substantial heterogeneity was observed, particularly in definitions of disease response, recurrence, and adverse events. For example, across 18 studies reporting ‘complete response’, definitions varied in assessment modality (clinical assessment alone, histology alone, cytology alone, or combined clinical–histological criteria), stringency (ranging from absence of visible lesions to absence of HSIL, LSIL, or any dysplasia), scope (lesion-specific *versus* field-wide assessment), and timing, which ranged from 3 weeks to 18 months post-treatment. Only three studies used validated patient-reported outcome measures (PROMs) for quality of life or functioning.

**Conclusion:**

Outcome reporting in anal HSIL treatment studies is highly inconsistent, limiting the synthesis of evidence and comparative analysis. These findings support the urgent need for a COS to standardize outcome selection and improve the quality and comparability of future trials.

## Teaser

This systematic review analysed 67 studies of treatments for anal high-grade squamous intraepithelial lesions (HSIL) and identified 966 reported outcomes, which were consolidated into 170 standardised outcome terms. Reporting was highly heterogeneous, with wide variation in definitions of recurrence, response, and adverse events, and patient-reported outcomes were rarely assessed. These findings highlight the urgent need for a core outcome set (COS) to standardise trial design, enable robust comparison across studies, and ensure outcomes are clinically meaningful and patient-centred.

## Introduction

Anal high-grade squamous intraepithelial lesion (HSIL), formerly termed anal intraepithelial neoplasia (AIN) II and III, is a human papillomavirus-associated precursor of anal squamous cell carcinoma (ASCC)^1^. The incidence of ASCC has increased significantly in several countries over recent decades^2,3^ and is projected to rise further^4,5^. Anal HSIL is more common among people living with HIV (PLWH), men who have sex with men, women with a history of lower genital tract neoplasia, and other immunosuppressed groups, but also occurs in the general population^6^. Lesions may cause non-specific symptoms such as bleeding, pruritus, pain, or a palpable abnormality^7^, but are often asymptomatic and detected through screening, after diagnosis of intraepithelial neoplasia elsewhere in the genital tract^8^, or incidentally following assessment for other anorectal conditions^9^. Left untreated, anal HSIL can progress to invasive anal cancer, with rates reported as high as 14.1% at 5 years in PLWH^10^. When symptomatic, anal HSIL may also cause troublesome morbidity, making it an important target for intervention.

Several treatment approaches for anal HSIL exist, including ablative therapies such as electrocautery ablation, infrared coagulation, radiofrequency ablation and laser ablation; topical agents such as imiquimod and 5-fluorouracil; and surgical excision, with other modalities also reported^11^. The landmark ANCHOR trial^12^, a multicentre phase III randomised controlled trial of treatment versus active surveillance for anal HSIL, in which electrocautery ablation predominated, demonstrated that treatment of anal HSIL in screened HIV-positive individuals halved the risk of malignant transformation to ASCC. These findings provide proof of principle that treating anal HSIL can prevent invasive anal cancer.

The next steps will be evaluation of different treatment modalities, broader populations, and people with differing disease extents. However, interpretation and comparison of existing studies are hampered by heterogeneous outcome definitions and inconsistent reporting, which limit evidence synthesis and weaken trial conclusions^13^. High-quality interventional studies that measure clinically meaningful outcomes are needed. A core outcome set (COS) is a key enabler. A COS provides a minimum set of outcomes that should be measured and reported in all trials for a given condition^14^. Developed with input from key stakeholders, implementation of a COS will standardise outcome reporting, enable robust comparison across trials, and ensure that outcomes are clinically meaningful to both patients and clinicians^14^.

As the first stage in COS development, this systematic review aimed to summarise the outcomes reported in studies evaluating treatments for anal HSIL.

## Methods

This systematic review was registered prospectively with the International Prospective Register of Systematic Reviews (PROSPERO CRD 42023429661) and is reported in accordance with the Preferred Reporting Items for Systematic Reviews and Meta-Analyses (PRISMA) guidelines^15^.

### Search strategy

MEDLINE, Embase, The Cochrane Library (Cochrane Database of Systematic Reviews, Cochrane Central Register of Controlled Trials (CENTRAL)), and Cumulative Index to Nursing and Allied Health Literature (CINAHL) databases were searched from their inception until June 2025 to identify studies evaluating any treatment in patients with anal HSIL. A full search strategy was developed with the support of a librarian from the University of Manchester. An example search algorithm for the MEDLINE database via PubMed is presented in the Supplementary Materials, *Table S1*. Reference lists of included studies were hand-searched for additional relevant studies.

### Eligibility criteria

Peer-reviewed systematic reviews, RCTs, prospective and retrospective cohort studies in adult populations (≥18 years), evaluating any treatment intervention for anal HSIL (AIN3 or p16-positive AIN2, or equivalent classifications such as high-grade AIN or carcinoma in situ), with full text available in English, were eligible for inclusion. Qualitative studies were eligible as a potential source of outcomes important to patients. The review’s inclusion and exclusion criteria are presented in *Table 1*.

**Table 1:** Inclusion and exclusion criteria for studies in the review

	Included	Excluded
Types of studies	➢ Systematic reviews ➢ Randomised/non-randomised controlled trials ➢ Early phase clinical trials ➢ Pilot studies ➢ Cohort studies (prospective or retrospective) ➢ Qualitative studies that relate specifically to aHSIL treatment outcomes. ➢ English-language studies	➢ Case-control studies ➢ Cross-sectional studies ➢ Case series ➢ Case reports ➢ Conference abstracts, editorials and expert opinion ➢ Non-English-language studies
Condition of interest	➢ Anal HSIL only (AIN3 or P16 positive AIN2)* ➢ Anal HSIL and anal LSIL (AIN1 or P16 negative AIN2)	➢ Anal LSIL only ➢ Anal condylomata (and anal HSIL) ➢ Extramammary Pagets/Anal intraepithelial adenocarcinoma (and anal HSIL) ➢ Vulva, Vagina, Cervix, Penile, Scrotal SIL (and anal HSIL)
Population	➢ Participants aged 18 years and over	➢ Participants below the age of 18
Interventions	➢ Any direct intervention (for example surgical excision, electrocautery/laser ablation, topicals (Imiquimod, 5-Fluorouracil, Trichloroacetic acid) and/or indirect interventions (for example therapeutic vaccines targeting high-risk HPV (hrHPV))	➢ No specific intervention exclusions
Outcomes	➢ Any reported treatment outcomes	➢ No treatment outcomes reported

*Where LAST classification/terminology not used: AIN2 alone, high-grade AIN (HGAIN), Perianal Bowen’s and anal carcinoma-in-situ, accepted as alternative to anal HSIL. AIN, Anal intraepithelial neoplasia; HPV, Human Papilloma Virus; HSIL, high-grade squamous intraepithelial lesion; LSIL, low-grade squamous intraepithelial lesion; SIL, Squamous intraepithelial lesion.

### Assessment of eligibility

Screening and selection of review articles were undertaken using Rayyan systematic review software^16^. Duplicate articles identified by the software were excluded. All remaining titles and abstracts were screened independently by two review authors. Any discrepancies were discussed and resolved between the two screening authors. Full-text screening of all included abstracts and titles was undertaken. For quality assurance, both authors initially screened the same five articles for inclusion; if they did not reach agreement, they screened a further five articles, and this process was repeated until agreement was reached. The remaining articles were then screened independently by another author. There were no unresolved disagreements that required escalation to the research team for a final decision.

### Data extraction

Data were extracted into a Microsoft Excel spreadsheet^17^. The following baseline characteristics were recorded for each study: author, title, journal, year of publication, country of the participating centre, study design, study intervention and regimen, number of participants, and characteristics of the population studied (that is participant age; grades of AIN treated; proportion of participants with anal HSIL; sex/gender; sexual behaviour or orientation; HIV status; location of treated lesions—perianal and/or anal canal; extent of disease), as well as the length of follow-up.

Any measurement or observation used to capture and assess the effect of the treatment was considered an outcome^14^. All outcomes reported by a study (and whether a primary outcome was stated) were extracted verbatim, along with any accompanying definition. Details of any outcome measurement methods/tools and the time point/period at which each outcome was measured were collected.

Synthesis of results and meta-analysis are beyond the scope of this review, which aims to identify which treatment outcome measures are being reported. Consequently, the methodological quality and risk of bias in the included studies were not assessed.

### Outcomes of interest

Outcomes specified in systematic reviews were extracted to account for the possibility that authors of systematic reviews might specify novel outcomes (for example a new composite outcome derived from individual outcomes reported in the existing literature). Articles reporting long-term results of eligible studies were included alongside the original publication to ensure that any additional outcomes were captured. For health-related quality of life (HRQoL) outcomes measured with HRQoL instruments, individual outcomes within each tool were extracted according to principles established by Macefield^18^.

Verbatim outcomes which had been worded, defined, or measured in different ways but ultimately had shared meaning were grouped together under a standardized outcome term (SOT) by a single researcher (DF). Each SOT was categorized using a taxonomy developed specifically by the COMET (Core Outcome Measures in Effectiveness Trials) initiative for classifying outcomes measured in clinical trials^19^. Under this taxonomy, each of the SOT were assigned to one of 38 domains, distributed across five core areas: *Death, Physiological/clinical, Life impact, Resource use, and Adverse events.*

The SOT and domain assigned to each verbatim outcome were reviewed and agreed upon at a meeting of the COrSIcA Study Steering Group (SSG), which comprised experts in the field of anal HSIL, including colorectal surgeons, gynecologists, and an HIV clinician, as well as an expert in COS methodology and a patient representative.

### Statistics

Descriptive statistical analysis was performed using Microsoft Excel^17^. The number and proportion of studies reporting each SOT were summarised using frequencies and percentages.

## Results

### Included studies

**Fig. 1:**
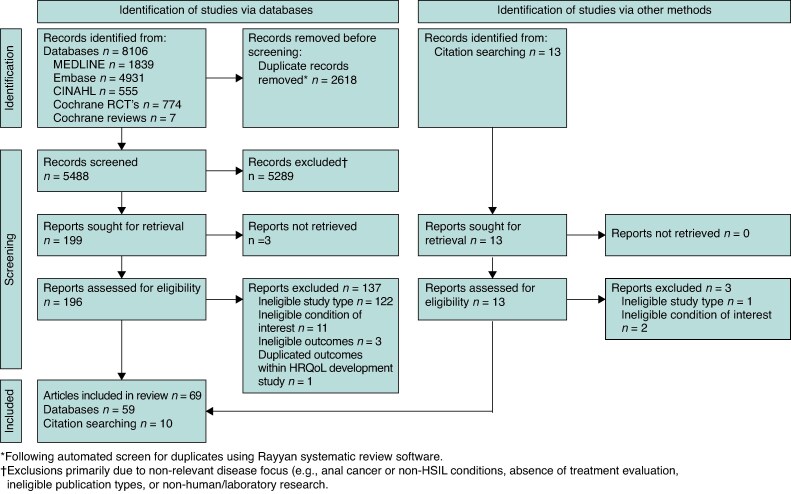
PRISMA flow diagram illustrating the identification, screening and inclusion of studies evaluating treatment interventions for anal HSIL

Searches in MEDLINE (via PubMed), Embase, Cumulative Index to Nursing and Allied Health Literature (CINAHL), and The Cochrane Library were conducted in June 2025, identifying 8106 records (MEDLINE: 1839, Embase: 4931, CINAHL: 555, Cochrane Library: 781). After removing duplicates, 5488 records were screened by title and abstract, of which 199 records were identified for retrieval. Full-text screening was conducted on 196 articles to determine eligibility, with 59 meeting the inclusion criteria. A further 13 articles were identified through citation searching, of which ten were included after full text review, bringing the total of articles included in the review to 69. A mixed-methods qualitative study identified during the search described the development of an HRQoL tool and its associated outcomes^20^. As this tool was later used in another study^21^, the qualitative study was excluded to prevent duplication. The screening process, including reasons for exclusion, is presented in the PRISMA flow diagram (*Fig. 1*).

### Study characteristics

The 69 articles included in this review represent 67 individual studies. One study reported findings across two articles^22,23^, another reported HRQoL findings of a subset of participants in a separate article^12,21^. Two studies that reported intermediate- and long-term follow-up^24,25^ of participants from earlier studies^26,27^ were retained as separate studies for the purposes of outcome evaluation.

Of the 67 included studies, 40 were observational (retrospective or prospective cohort), 23 were interventional (of which 5 were RCTs), and 4 were systematic reviews. Just eleven studies involved comparisons with other treatment modalities or non-treatment strategies.

Across the included studies, 17 treatment types were identified; the most commonly reported was electrocautery ablation, used in 18 studies. A comprehensive overview of study characteristics for the 67 included studies is provided in Supplementary Materials, *Table S2*, with a concise summary presented in *Table 2*.

**Table 2:** Characteristics of the 67 included studies evaluating treatment intervention for participants with anal HSIL

Characteristic	Characteristic subcategory	Number of studies (%)
Study type	Retrospective cohort study*	33 (49)
	Prospective cohort study	7 (10)
	Pilot/Early phase clinical trial	18 (27)
	Randomised controlled trial	5 (7)
	Systematic review	4 (6)
Number of participants†	<50	32 (51)
	≥50 to <100	18 (29)
	≥100 to <200	7 (11)
	≥200	6 (10)
Mean/median age†	≥30 to <40	41 (65)
	≥40 to <50	13 (21)
	≥50 to <60	4 (6)
	Not described	5 (8)
Sex (% male)†	0	1 (2)
	1–25	0 (0)
	>25–75	10 (16)
	>75–<100	13 (21)
	100	15 (24)
	Not specified	4 (6)
HIV status (% HIV + ve)†	0	5 (8)
	1–25	2 (3)
	>25–75	7 (11)
	>75–<100	5 (8)
	100	39 (62)
	Not specified	5 (8)
MSM status of male participants†	0	0 (0)
	1–25	0 (0)
	>25–75	2 (3)
	>75–<100	4 (6)
	100	31 (49)
	Not specified	25 (40)
	Not applicable	1 (2)
Disease location†	Canal only	36 (57)
	Perianus only	3 (5)
	Canal and perianus	20 (46)
	Not specified	4 (6)
Treatment type†	Surgical excision‡	11 (17)
	Electrocautery ablation	18 (29)
	Laser ablation	6 (10)
	Radiofrequency ablation	4 (6)
	Infrared coagulation	17 (27)
	Argon plasma coagulation	1 (2)
	Photodynamic therapy	2 (3)
	Imiquimod	11 (17)
	5-Fluorouracil	6 (10)
	Cidofovir	4 (6)
	Cryotherapy	1 (2)
	Trichloroacetic acid	7 (11)
	Therapeutic vaccination	3 (5)
	Topicals (not specified)	1 (2)
	Artesunate suppositories	1 (2)
	Endoscopic excision	2 (3)
	Sinecatechins	1 (2)
	Active monitoring	4 (6)

^*^Including retrospective studies of prospectively collected data. †(*n*, number of studies = 63 (excluding 4 systematic reviews comprising individual studies already captured by the review). ‡Including wider excision with split-skin graft/flap reconstruction. MSM, Men who have sex with men.

### Outcomes reported

**Fig. 2:**
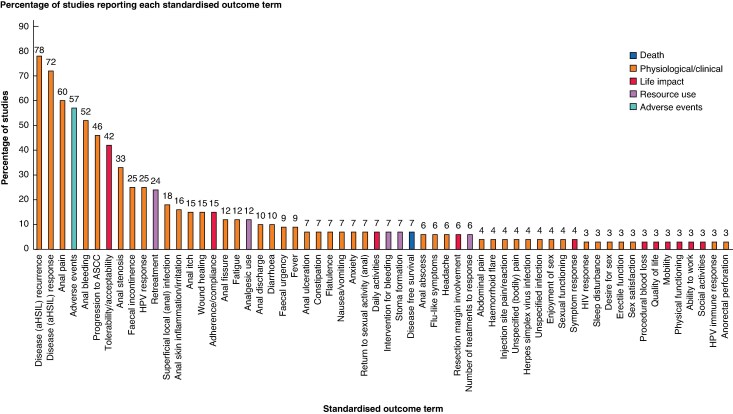
Frequency of SOTs reported in anal HSIL treatment studies for the 60 most frequently reported SOTs, colour-coded by core area

A total of 966 outcomes were reported across the 67 studies, of which 573 (59%) were accompanied by a definition. There were 568 unique verbatim outcome terms (VOTs). After accounting for outcomes worded differently but with the same or similar meaning, the outcomes collapsed to 170 SOTs. *Figure 2* shows the frequency of SOTs reported for the 60 most frequently reported SOTs. A ‘primary’ or ‘main’ outcome was described in 29/67 (43%) of studies. SOTs were identified across all five core areas and in 31/38 outcome domains of the COMET outcome taxonomy, with Physiological/clinical outcomes most frequently represented (131/170 SOTs; 77%). A full breakdown of all verbatim outcomes and SOTs by outcome domain and core area is presented in Supplementary Materials, *Table S3*.

#### Core area: death

The core area *Death* comprises a single outcome domain, *Mortality/Survival.* Six SOTs were categorised under this core area: death (reported in 2/67 (3%) studies), death from metastatic anal cancer (1/67, 1%), death unrelated to treatment effect (1/67, 1%), disease-free survival (5/67, 7%), overall survival (1/67, 1%) and treatment related death (2/67, 3%).

#### Core area: physiological/clinical

A total of 131 SOTs were categorized under the core area, *physiological, or clinical*. Outcomes within this core area encompassed 19 of the 23 outcome domains. *Outcomes relating to neoplasm* were the most frequently reported domain, featuring in 66/67 (99%) of studies. The domain with the greatest number of outcomes was *Gastrointestinal*, with 35 outcomes. Seven of the ten most frequently reported outcomes fell within this core area: disease recurrence (52/67 (78%) studies), disease response (48/67, 72%), anal pain (40/67, 60%), anal bleeding (35/67, 52%), progression to ASCC (31/67, 46%), anal stenosis (22/67, 33%) and faecal incontinence (17/67, 25%) (*Fig. 2*).

Outcomes relating to neoplasm were reported in 66/67 (99%) studies; however, there was marked heterogeneity of the verbatim terms and definitions used. For example, the verbatim outcome ‘Complete response’ was reported in 18 studies. Definitions varied considerably from a strict criterion of no dysplasia at any location over two consecutive time points to a more lenient definition that accepted low-grade squamous intraepithelial lesions (LSIL) as a complete response^28,29^. Assessment methods also differed: some studies relied solely on clinical examination^30^, while others required confirmation through cytology^31^, histology^22^, or both cytology and histology^28,29,31^. In some studies, evaluation was lesion-specific; in others, field-wide, with biopsies taken from both treated and newly detected lesions^32,33^. The timing of assessment was inconsistent, ranging from 4–8 weeks to 18 months, and few studies reported blinding of clinical or pathology reviewers. Only one study reported a response outcome based on changes in the amount of anal HSIL^29^. *Table 3* presents the criteria used to define the verbatim outcome term ‘complete response’ across included studies^13,22,27–42^.

**Table 3:** Criteria used to define the verbatim outcome term ‘complete response’

Study	Verbatim definition	Approach assessment	Scope of assessment	Criteria	Timing of assessment
B Klencke 2002^34^	Complete absence of dysplastic cells in the biopsy specimens at two consecutive points	Anal Pap smear + HRA +/− biopsy	Field-wide (biopsies of suspicious areas at HRA regardless of whether they were at the treated site or new)	Cytology ALONE (if no clinically suspicious lesions) showing complete absence of dysplastic cells	12 and 24 weeks
				Cytology AND Histology (if suspicious lesion) showing complete absence of dysplastic cells	
JM Palefsky 2006^31^	Regression to no AIN	Anal Pap smear + HRA +/− biopsy	Field-wide (biopsies were taken of any lesion suspicious for HSIL)	Cytology ALONE (if no clinically suspicious lesions) showing complete absence of AIN	8, 12, 24 and 48 weeks
				Cytology AND Histology (if suspicious lesion) showing complete absence of AIN	
U Wieland 2006^27^	Clinical and histologic clearance of anal intraepithelial neoplasia (AIN) at the end of therapy	HRA guided clinical assessment + biopsy	Lesion-specific (biopsies were taken adjacent to the site of the original lesion)	Clinical (no visible lesion at treated site) AND Histology (showing no AIN)	End of treatment (16 weeks)
EA Stier 2008^28^	Normal histology or low-grade squamous intraepithelial lesion (LSIL) on biopsy, combined with normal or minimally abnormal cytology (no HSIL) at follow-up	HRA guided biopsy of treatment sites + Anal Pap smear	Lesion-specific (see comment) (if no abnormal lesion was visible, a biopsy was still taken at the treated sites)	Cytology AND Histology showing either normal or LSIL.	3, 6, 9 and 12 months
O Richel 2010^35^	Clinical and histological resolution of AIN	HRA guided clinical assessment and biopsies	Lesion-specific (biopsies taken from the same location as previously diagnosed lesions)	Clinical AND Histological resolution	4 weeks completing treatment
EA Stier 2013^29^	Absence of any high-grade Perianal AIN or cancer.	Acetic acid guided clinical assessment and biopsies	Lesion-specific (assessment focused on previously photo-documented and biopsied lesions (residual or original site)).	Clinical AND Histological absence of high-grade disease	6 weeks post treatment
O Richel 2013^22^	Resolution of AIN	HRA guided biopsy	Field-wide (previously treated and newly suspicious lesions)	Histological resolution of AIN	4 weeks post treatment
EM Van der Snoek 2015^36^	Reduction of moderate or severe dysplasia (HSIL) to complete absence of dysplasia.	HRA guided biopsy	Lesion-specific (biopsies taken from previously diagnosed HSIL areas only)	Histological absence of dysplasia (no LSIL or HSIL)	3 weeks post completion of treatment
J Burgos 2016^37^	Resolution of AIN in the biopsy sample obtained after the end of treatment	HRA guided biopsy	Lesion-specific (biopsy was taken from the previously treated areas, regardless of whether they looked normal at HRA)	Histological resolution of AIN	6–8 weeks post treatment
ML Siegenbeek van Heukelom 2018^30^	if no lesions were seen, treatment response was defined as a complete response (CR) … If histological resolution of all AIN lesions was achieved, treatment response was also defined as CR.	HRA guided clinical inspection (visual inspection).@+/− biopsies (if any lesions were visible).	Lesion-specific (visual and biopsy assessments targeted previously known or treated lesions)	Clinical (no visible lesions) OR Histological visible lesion histological (no AIN on biopsy)	4–6 weeks post treatment
J Burgos 2018^38^	Resolution of SIL	HRA guided biopsy	Lesion-specific (Biopsies were taken from previously treated areas, even if the area appeared normal at HRA)	Histological resolution of AIN	6–8 weeks post treatment
D Brogden 2021^13^	High- or low-grade AIN that on follow-up after treatment no AIN of any grade persists.	N/A	N/A	N/A	N/A
I Fuertes 2021^39^	An absence of any dysplasia.	HRA guided biopsy	Field-wide (biopsies were taken at the index site regardless of macroscopic abnormalities, and at any new suspicious sites detected by HRA)	Histological absence of any dysplasia	18 months post treatment
I Fuertes 2022^40^	Patients without clinical signs of HSIL in the previously treated area	HRA guided clinical inspection (visual inspection).@+/− biopsies (if any lesions were visible).	Lesion-specific (visual and biopsy assessments targeted previously known or treated lesions)	Clinical (no visible lesions) OR Histological if visible lesion (no AIN on biopsy)	1–6 months post treatment
J Burgos 2023^33^	Resolution of HSIL up to normal on the post treatment biopsy sample.	HRA guided biopsy	Field-wide (biopsies were taken from the areas where HSIL had previously been reported, even if they looked normal.@- Biopsies were also taken from any new suspicious lesions seen at HRA)	Histological absence of any dysplasia	6–8 weeks post treatment
KCM Gosens 2023^32^	Disappearance of lesions as assessed by HRA, confirmed by biopsy of the former lesion site, or, if lesions persisted, histologic resolution of AIN.	HRA guided biopsy	Field-wide: (Patients without residual HSIL lesions, including both treated and new lesions, at follow-up were considered to have a complete response).	Histological absence of any dysplasia	3, 6- and 12-months post treatment
J Burgos 2025^41^	Absence of squamous intraepithelial lesions (SIL) on biopsy	HRA guided biopsy	Field-wide: an anal biopsy was performed in areas where HSIL was previously reported and, if necessary, from other suspect lesions.	Histological absence of any dysplasia	10 weeks post completion of treatment
N Gallio 2025^42^	Complete response (CR) was defined as histological or cytological evidence of total regression, and in the absence of these criteria, on clinical examination.	N/A	N/A	N/A	N/A

Inferred from modality of assessment (Anal cytology by method of collection not specific to a treated area).

#### Core area: life impact

A total of 22 outcomes were identified across the eight domains comprising the *Life Impact* core area. The *Delivery of care* domain was the most frequently represented and also contained the most common SOT for this core area: tolerability/acceptability (of treatment), reported in 28/67 (42%) of studies.

In contrast, outcomes relating to the six functioning and quality of life (QoL) domains were represented in only a few studies overall: *Physical functioning* (5/67 studies, 7%), *Emotional functioning/wellbeing* (4/67, 6%), and *Cognitive functioning, Role functioning, and Social functioning* (each 2/67, 3%). Outcomes related to sexual functioning, categorized under the *Reproductive outcomes* domain within the *Physiological/clinical* core area but considered here from a functioning perspective, were reported in 9/67 (13%) of studies. Of the 14 treatment studies reporting outcomes in these domains, three used a validated patient-reported outcome measure (PROM) to assess functioning or QoL^21,23,43^. A full breakdown of SOTs extracted from PROMs assessing functioning and QoL is detailed in Supplementary Materials, *Table S4*.

#### Core area: resource use

In the core area *Resource use*, nine of the ten outcomes are related to a single domain—*Need for further intervention*. The most commonly reported SOT was retreatment (for persisting or recurrent disease), reported in 16/67 (24%) of studies (see Supplementary Materials, *Table S3* for a full breakdown of outcomes reported within this core area).

#### Core area: adverse events

An adverse event outcome (either a broadly labeled summary outcome of adverse events for example ‘Adverse effects’, ‘Side effects’, or ‘Complications’, and/or specifically named adverse events for example ‘Anal pain’, ‘Anal bleeding’, or ‘Anal infection’) was reported in 59/67 (88%) of included studies.

35/67 (52%) studies reported both a summary outcome of adverse events and specifically named adverse events; 22/67 (33%) studies reported only specifically named adverse events, and 2/67 (3%) studies reported only a summary of adverse events.

Summary outcomes of adverse events ranged in their descriptions from simply reporting the occurrence of adverse events to more detailed outcomes characterized by adverse event type, severity, duration, or time to onset, but without information on the body system(s) involved.

At least one specifically named adverse event was reported in 57/67 (85%) studies.

SOTs for the five most common specifically named adverse events were anal pain (40/67 (60%) studies), anal bleeding (35/67 (52%) studies), anal stenosis (22/67 (33%) studies), faecal incontinence (17/67 (25%) studies) and superficial local (anal) infection (12/67 (18%) studies).

Only 14/67 (21%) studies reported adverse events using a validated grading system. The Common Terminology Criteria for Adverse Events (CTCAE) was used in 9/67 (13%) studies, the Division of AIDS Table for Grading the Severity of Adult and Paediatric Adverse Events in 3/67 (4%) studies, and the Clavien-Dindo classification in 2/67 (3%) studies. Nine studies (13%) differentiated adverse events unrelated to treatment from those directly attributable to treatment.

## Discussion

The practice-changing evidence provided by the ANCHOR trial, together with recently published international consensus guidelines recommending screening of populations at high risk for ASCC^44^, is expected to increase the number of patients undergoing treatment for anal HSIL. This, alongside the lack of consensus on optimal treatment approaches^13^, underscores the urgent need for well-designed interventional studies that measure clinically relevant, standardized outcomes to inform best practices in the treatment of anal HSIL.

This systematic review is the first step towards developing a COS for interventional studies in anal HSIL. The review presents a comprehensive examination of the outcomes used in current anal HSIL studies, mapped to a recognized outcome taxonomy. A critical finding of our review is the widespread heterogeneity in outcome definition and measurement. While frequently reported, even fundamental outcomes such as disease response exhibit considerable variation in the definitions used and in the assessment modalities and time points employed. This lack of standardization extends to the reporting of adverse events, where comprehensive categorization and consistent grading using validated systems such as CTCAE were observed in only a minority of studies.

The most frequently reported outcomes, including disease recurrence (78%), disease response (73%), anal pain (60%), anal bleeding (52%), and progression to ASCC (46%), reflect clinically relevant concerns. However, the inconsistent definitions applied to these outcomes significantly impede their utility for cross-study comparisons. For example, while recurrence is a key outcome, its definition can conflate true recurrence with persistence or new metachronous lesions, thereby affecting reported rates and making interpretation challenging^13^.

A notable gap identified by this review is the paucity of studies reporting outcomes related to functioning and QOL. Only three studies utilized a validated PROM to assess these crucial patient-important outcomes. This omission means that the impact of anal HSIL treatments on patients’ daily lives, emotional well-being, and sexual functioning is largely unquantified.

The strength of this review lies in its rigorous methodology. The comprehensive search strategy across multiple databases, inclusion of both interventional and observational studies, and systematic categorisation of outcomes using the COMET taxonomy enhance the credibility and reproducibility of our findings. This methodological approach allowed for a nuanced mapping of outcome heterogeneity, enabling clearer identification of priorities for future COS development. The collaborative approach to standardising outcomes with an expert steering group, including patient representation, ensures clinical relevance and patient-centredness.

Some limitations must be acknowledged. This study was designed to map the scope and nature of outcome reporting in anal HSIL treatment studies rather than to synthesise effect estimates; accordingly, formal assessment of methodological quality and risk of bias was not undertaken. Such assessments, while essential for meta-analyses, are less informative for a descriptive review of outcome measurement. Despite full concordance during an initial calibration exercise, study selection was undertaken by a single reviewer, and some risk of selection bias cannot be excluded. The review was restricted to full-text articles published in English due to resource limitations and the lack of readily available translation facilities. This may have excluded relevant studies published in other languages, potentially omitting outcome measures used in non-English-speaking settings. However, the 67 included studies represent research from thirteen countries, several of which are non-English-speaking, suggesting that the review captures a degree of global variation in outcome reporting. While some region-specific practices may still be under-represented, the international spread of included studies, together with the diverse expertise within our steering group, should help to minimise any impact on the global applicability of a future COS.

In conclusion, this systematic review has identified substantial heterogeneity in outcome reporting in anal HSIL treatment trials and highlights the urgent need for a COS in this disease area. The comprehensive list of outcomes identified in this review is the first step towards the development of this COS, the adoption of which will be instrumental in fostering high-quality, comparable and meaningful research that ultimately improves patient care for individuals affected by anal HSIL.

## Supplementary Material

zrag061_Supplementary_Data

## Data Availability

All data generated or analysed during this study are included in this published article and its *Supplementary Material* files. Additional data related to outcome extraction are available from the corresponding author upon reasonable request.
