# Supplementary material for: Outcomes reported in studies of anal high-grade squamous intraepithelial lesions treatments: systematic review
Source: BJS Open. 2026 Jul 4;10(4):zrag061. doi: 10.1093/bjsopen/zrag061 (PMC13332405; doi:10.1093/bjsopen/zrag061)
Supplement: zrag061_Supplementary_Data [file zrag061_supplementary_data.zip › Supplementary_Material.docx]

**Systematic Review of Outcomes Reported in Treatment Studies of Anal High-Grade Squamous Intraepithelial Lesions to Inform Core Outcome Set Development**

**Authors:** ^1^David A. Finch, ^2^Madhu P. Chaudhury, ^3^Rebecca Morris, ^3^Edward Parkin, ^3^Peter Mitchell, ^4^Pierre Martin-Hirsch, ^1,5^Andrew G. Renehan, ^5^Rebecca Fish.

**Author affiliations:** ^1^Division of Cancer Sciences, University of Manchester, United Kingdom. ^2^Department of Colorectal surgery, Lancashire Teaching Hospitals NHS Foundation Trust, United Kingdom. ^3^Division of Population Health, Health Services research and Primary Care, University of Manchester, United Kingdom. ^4^Department of Gynaecological oncology, Lancashire Teaching Hospitals NHS Foundation Trust, United Kingdom. ^5^Colorectal and Peritoneal Oncology Centre (CPOC), The Christie NHS Foundation Trust, United Kingdom.

**Correspondence to:**

David Finch

Room 2.020

Manchester Cancer Research Centre

Oglesby Building

555 Wilmslow Road

Manchester

M20 4GJ

t: +44(0)161 306 0800

e: david.finch-2@manchester.ac.uk

Supplementary Materials – Index

**Supplementary Methods:**

[Table S1. Medline search strategy 3](#_Toc218028999)

**Supplementary Figures and Tables:**

[Table S2. Treatment study characteristics and standardised outcome terms reported for each study. 5](#_Toc218029000)

[Table S3. List of verbatim outcomes and ‘standardised outcome terms’ by core area and outcome domain. 19](#_Toc218029001)

[Table S4. Outcomes extracted from quality of life/functioning patient-reported outcome measurement (PROM) tools. 36](#_Toc218029002)

[References 38](#_Toc218029003)

Table S1. Medline search strategy

Database: PubMed

Platform: NCBI

Date searched: 28/06/2023

Search strategy:

| **Search number** | **Query** |
| --- | --- |
| **1** | AIN[Title/Abstract] |
| **2** | anal intraepithelial neoplasia[Title/Abstract] |
| **3** | high grade squamous intraepithelial neoplasia[Title/Abstract] |
| **4** | HSIL[Title/Abstract] |
| **5** | high grade anal intraepithelial neoplasia[Title/Abstract] |
| **6** | HGAIN[Title/Abstract] |
| **7** | (((((#1) OR (#2)) OR (#3)) OR (#4)) OR (#5)) OR (#6) |
| **8** | treatment[Title/Abstract] |
| **9** | management[Title/Abstract] |
| **10** | therapy[Title/Abstract] |
| **11** | excision[Title/Abstract] |
| **12** | surgical excision[Title/Abstract] |
| **13** | ablation[Title/Abstract] |
| **14** | cautery[Title/Abstract] |
| **15** | electrocautery[Title/Abstract] |
| **16** | diathermy[Title/Abstract] |
| **17** | hyfrecation[Title/Abstract] |
| **18** | radiofrequency ablation[Title/Abstract] |
| **19** | infrared coagulation[Title/Abstract] |
| **20** | argon plasma coagulation[Title/Abstract] |
| **21** | laser[Title/Abstract] |
| **22** | imiquimod[Title/Abstract] |
| **23** | fluorouracil[Title/Abstract] |
| **24** | 5-FU[Title/Abstract] |
| **25** | trichloroacetic acid[Title/Abstract] |
| **26** | cidofovir[Title/Abstract] |
| **27** | photodynamic therapy[Title/Abstract] |
| **28** | sinecatechin*[Title/Abstract] |
| **29** | vaccin*[Title/Abstract] |
| **30** | (((((((((((((((((((((#8) OR (#9)) OR (#10)) OR (#11)) OR (#12)) OR (#13)) OR (#14)) OR (#15)) OR (#16)) OR (#17)) OR (#18)) OR (#19)) OR (#20)) OR (#21)) OR (#22)) OR (#23)) OR (#24)) OR (#25)) OR (#26)) OR (#27)) OR (#28)) OR (#29) |
| **31** | (#7) AND (#30) |
| **32** | vagina[Title] |
| **33** | vaginal[Title] |
| **34** | vulva[Title] |
| **35** | vulval[Title] |
| **36** | cervix[Title] |
| **37** | cervical[Title] |
| **38** | (((((#32) OR (#33)) OR (#34)) OR (#35)) OR (#36)) OR (#37) |
| **39** | (#31) NOT (#38) |
| **40** | anterior interosseous nerve[Title/Abstract] |
| **41** | allergic interstitial nephritis[Title/Abstract] |
| **42** | acute interstitial nephritis[Title/Abstract] |
| **43** | Al-AIN[Title/Abstract] |
| **44** | (((#40) OR (#41)) OR (#42)) OR (#43) |
| **45** | (#39) NOT (#44) |
| **46** | (#39) NOT (#44) |

Notes: English language filter

Table S2. Treatment study characteristics and standardised outcome terms reported for each study.

| **Author** | **Year** | **Country** | **Study design** | **Intervention and regimen** | **Number of participants** | **Age** | **AIN grades treated** | **% with anal HSIL** (AIN ≥ 2, PBD, CIS, HSIL, HGAIN) | **Sex** | **Men who have sex with men** | **HIV +ve (%)** | **Location of treated disease** | **Disease extent** | **Follow-up duration** | **Standardised outcome terms** (Primary outcome in **bold**, if stated) |
| --- | --- | --- | --- | --- | --- | --- | --- | --- | --- | --- | --- | --- | --- | --- | --- |
| **J Scholefield[1]** | 1994 | UK | Retrospective cohort study (chart review) | **Surgical excision +/- split skin grafting** (for lesions occupying >50% anal canal/perianus). | 27 (6 undergoing split skin grafting) | Median age 40 (range 30-77) | HGAIN | 100 | Not specified | Not specified | Not specified | Canal and/or Perianus | Lesions <1 cm and >1 cm in diameter (2 participants had canal lesions <1 cm; 25 participants had perianal lesions: 10 had lesions >1 cm, and 15 had lesions <1 cm). | Median 20 months (range 1-43) | Anal stenosis  Closure of stoma  Faecal incontinence  Recurrence  Stoma formation  Wound healing |
| ***P Marchesa[2]** | 1997 | USA | Retrospective cohort study (chart review) | **Wide local excision (WLE)** (with 1cm macroscopic clearance) **+/- split skin graft or advancement flap** vs **Local excision** vs **CO2 Laser.** | 47 (26 WLE (with 1cm macroscopic clearance) +/- split skin graft or advancement flap; 15 Local excision; 5 CO2 Laser | Median age 46 (range 46-90) | Perianal Bowen’s disease (PBD) | 100 | 34% male  (16M:31F) | Not specified | Not specified | Canal and/or Perianus | Not specified | Median 104 months (range 16-273) | Death unrelated to treatment effect  Overall survival  Progression to ASCC  **Recurrence** |
| **S Brown[3]**  ****** | 1999 | UK | Retrospective cohort study (chart review) | **Surgical excision +/- split skin grafting/mucosal advancement flap** (for lesions occupying >50% anal canal/perianus). | 34 | Not described | HGAIN | 100 | Not specified | Not specified | Not specified | Canal and/or Perianus | Lesions <1 cm (n=15) and >1 cm (n=19) in diameter; lesions >1 cm further stratified by <50% (n=9) and >50% (n=10) anal circumference. | Median 41 months (range 12-104) | Adverse events  Anal stenosis  Faecal incontinence  Recurrence  Retreatment  Stoma formation |
| **B Klencke[4]** | 2002 | USA | Early phase trial (Phase 1) | **Therapeutic vaccine ZYC101:** 4 doses at 3 weekly intervals. | 12 | Mean age 45 (range 27-68) | 2,3 | 100 | 100% male | 100% MSM | 0 | Canal and/or Perianus | Not specified | 24 weeks | Adverse events  Fatigue  Fever  Headache  HPV immune response  HPV response  Injection site pain/reaction  Response  Sweats  Thigh Erythema  Tolerability/acceptability of intervention |
| **GJ Chang[5]** | 2002 | USA | Prospective cohort study | **Electrocautery ablation:** single treatment session unless circumferential disease then a staged approach with second procedure 3 months after first procedure. | 37 | Mean age 45 (+/- 8 years) | 2,3 | 100 | 100% male | Not specified | 78% HIV +ve | Canal and/or Perianus | Not specified. Although treatment approach inclusive of participants with circumferential disease (see regimen). | Mean 32.3 +/- 20.6 months (HIV -ve); 28.6 +/- 12.9 months (HIV +ve) | Anal abscess  Anal bleeding  Anal pain  Anal stenosis  Disease free survival  Enjoyment of sex  Faecal incontinence  Intervention for bleeding  Recurrence  Reoperation for abscess  Retreatment  Return to sexual activity (anal)  **Tolerability/acceptability of intervention** |
| **J Webber[6]** | 2004 | USA | Early phase trial | **Photodynamic therapy:** single session treatment to the entire anal circumference. | 5 | Not specified | Carcinoma in situ (CIS) | 100 | 100% male | Not specified | 100% HIV +ve | Canal | Not specified | 5 months | Anal pain  Anal stenosis  Blood cell count  Blood pressure  Blood sugar  Coagulation  Kidney function  Liver function  Nausea/vomiting  Platelets  **Response** |
| **SE Goldstone[7]** | 2005 | USA | Retrospective cohort study (chart review) | **Infrared photocoagulation (IRC) ablation:** single session ablation under LA. Further IRC offered for new or persistent HSIL. | 68 | Median age 41 (range 29-62) | HSIL | 100 | 100% male | 100% MSM | 100% HIV +ve | Canal | Discrete, non-circumferential disease only; circumferential or bulky disease excluded. Mean: 1.6 lesions per patient (range 1-5). | Median 203 days (range 162-1313) | Anal bleeding  Anal pain  Anal stenosis  Progression to ASCC  Recurrence  Retreatment  Return to sexual activity (anal)  Superficial local (Anal) infection  Tolerability/acceptability of intervention |
| **J Scholefield[8]** | 2005 | UK | Retrospective cohort study (prospectively collected data) | **Surgical excision** | 35 | Median age 43 (range 31-62) | 3 | 100 | 26% male  (9M:26F) | Not specified | 0 | Canal and/or Perianal (mostly perianal, some lesions extending into canal. One with isolated canal disease) | Multifocal (7 patients) or Monofocal (28 patients). All lesions occupying no more than 30% of the circumference of the anal area. | Median 63 months (range 14-120) | Adverse events  Death from metastatic anal cancer  Recurrence  Resection margin involvement  Retreatment  Superficial local (Anal) infection |
| **BD Graham[9]** | 2005 | USA | Prospective cohort study | **Topical 5% 5-FU:** for extensive disease (>50% circumference), applied BD for 16 weeks. **Local excision:** for localized disease (≤3.5 cm lesions). Further treatment with 5-FU or surgery at 16 weeks for residual disease. | 11 (8 5-FU; 3 Local excision) | Mean age 48 (range 32-75) | Bowen’s disease | 100 | 45% male  (5M:6F) | Not specified | 9% HIV +ve (1 participant) | Canal and/or Perianus (9 participants perianal; 2 canal and perianal) | Three patients had lesions <3.5 cm. Eight patients had >50% of the anoderm affected, of whom five had >75% involvement. | Mean 39 months (range 12-74) | Anal scarring  Anal sensation  Anal skin pigment change  Anoderm thinning  Recurrence  Resection margin involvement  Response  Retreatment  Small telangiectasia  Symptom response  Wound healing |
| **JM Palefsky[10]** | 2006 | USA | Early phase trial | **Therapeutic HPV Vaccine SGN00101:** 3 doses at 4 weekly interval. | 15 | Mean age 47.5 | 2,3 | 100 | 87% male  (13M:2F) | Not specified | 100% HIV +ve | Canal and/or Perianus | Not specified | 48 weeks | Adverse events  CD4 T cell count response  CD8 T cell count response  Dizziness  Fever  HIV response  HPV response  Injection site pain/reaction  Response  Rigors and chills  Sleep disturbance  Sweats |
| **U Wieland[11]** | 2006 | Germany | Pilot clinical trial | **5% Imiquimod:** cream or suppositories, three times weekly for 16 weeks. | 28 | Mean age 43 (range 31-69) | 1,2,3 | 64 | 100% male | 100% MSM | 100% HIV +ve | Canal and/or Perianus (23 participants perianal; 5 canal) | 12 participants monofocal lesions; 16 multifocal. | Mean 9.5 months (range 1-20) | Adherence/Compliance  Adverse events  Anal skin inflammation/irritation (generalised)  Anal ulceration  Flu-like symptoms  HPV response  Recurrence  **Response**  Tolerability/acceptability of intervention |
| **AJM Watson[12]** | 2006 | New Zealand | Retrospective cohort study (prospectively collected data) | **Perianal skin excision** | 72 | Median 49 (range 18-81) | 1,2,3 | 76 | 28% male  (20M:52F) | Not specified | 7% HIV +ve | Perianal | Not specified | Median 60 months (range 18-112) | Faecal incontinence  Progression to ASCC  Response  Response  Stoma formation |
| **SE Goldstone[13]** | 2007 | USA | Retrospective cohort study (chart review) | **Infrared Coagulation (IRC):** single session ablation under LA with further sessions for ongoing disease during follow up. | 75 | Median age 36 (range 20-71) | HSIL | 100 | 100% male | 100% MSM | 0 | Canal | Median number of lesions treated per participant was 1.5 (range 1-3). Patients with extensive disease were excluded. | Median 575 days (range 169-1499) | Adverse events  Anal bleeding  Anal pain  Anal stenosis  Analgesic use  Number of treatments to response  Progression to ASCC  Recurrence  Retreatment  Return to sexual activity (anal)  Superficial local (Anal) infection  Wound healing |
| **CE Pineda[14]** | 2007 | USA | Retrospective cohort study (prospectively collected data) | **Electrocautery alone;** **IRC +/- TCA:** in staged procedures for circumferential disease; **Active monitoring**. Patients with recurrences amenable to HRA-guided office-based procedures were treated with either IRC or TCA. | 42 (29 ECA; 4 IRC +/- TCA; 9 AM) | Male mean age 39 (range 21-63); Female mean age 50, range 31-71) | LSIL, HSIL | 79 | 71% male  30M:12F | Not described | 0 | Canal and/or Perianus | 4 participants with at least circumferential disease. No further details on extent specified. | Mean 36 months (range 5-125 months) | Adverse events  Anal fissure  Anal stenosis  Death  Flap reconstruction  Progression to ASCC  Recurrence  Response  Retreatment  Stoma formation  Superficial local (Anal) infection |
| **M Nathan[15]** | 2008 | UK | Retrospective cohort study (chart review) | **Imiquimod only; Excision only; Laser only; Imiquimod and excision; Imiquimod and laser; Excision and laser; Imiquimod and excision and laser; No treatment.** | 181 (10 Imiquimod only; 21 Excision only; 72 Laser only; 3 Imiquimod and excision; 38 Imiquimod and laser; 19 Excision and laser; 12 Imiquimod and excision and laser; 6 No treatment. | Mean age 34.6 years (range 19.3-61.7) | 1,2,3 | 49 | 99% male  (180M:1 Unknown) | 85% MSM | 46 (84 participants) | Not specified | Number of quadrants affected:  1 quadrant: 44 (24.3%);  2 quadrants: 43 (23.8%);  3 quadrants: 63 (34.8%);  4 quadrants: 30 (16.6%). | Median 19 months (range 3-26) | Anal pain  Daily activities  **Response**  Tolerability/acceptability of intervention |
| **RD Cranston[16]** | 2008 | USA | Retrospective cohort study (unspecified data collection) | **Infrared Coagulation (IRC):** single session ablation under LA | 68 | Median 46 (range 28-65) | 2,3 | 100 | 100% male | 100% MSM | 100 | Canal | Single lesions that extended to no more than half of the anal canal circumference. Four patients had two areas of high-grade disease and two had three areas of high-grade disease treated. | Mean 140 days (range 37-831) | Anal bleeding  Anal pain  Response  Superficial local (Anal) infection  Tolerability/acceptability of intervention |
| **A Kreuter[17]**  ******* | 2008 | Germany | Prospective cohort study | **5% Imiquimod:** cream or suppositories applied three times weekly for 16 weeks. | 19 | Not described | 1,2,3 | 68 | 100% male | 100% MSM | 100 | Canal and/or Perianus (16 participants perianal; 3 participants canal) | Not specified | Mean 30 months (range 11-39) | HPV response  Recurrence  Response |
| **CE Pineda[18]** | 2008 | USA | Retrospective cohort study (chart review) | **Electrocautery; IRC; TCA:** Single session or staged treatment for circumferential or near-circumferential lesions. | 246 | Mean male age 44 (range 24-63); Mean female age 44 (range 26-71) | HSIL | 100 | 84% male (207M:39F) | Not described | 74% HIV +ve (182 patients) | Canal and/or perianus (92 patients Canal; 3 Perianal; 125 Both. | Limited disease (<25% anal circumference): 46 patients (19%); Extensive disease (25-75% anal circumference): 168 patients (68%); Circumferential disease (>75% anal circumference): 32 patients (13%). | Mean 41 months (2-125) | Adverse events  Anal bleeding  Anal fissure  Anal stenosis  Disease free survival  Faecal incontinence  Myocardial infarction  Progression to ASCC  Recurrence  Retreatment  Superficial local (Anal) infection |
| **EA Stier[19]** | 2008 | USA | Pilot clinical trial | **Infrared coagulation (IRC):** single session ablation under LA. | 18 | Mean age 44 (range 32-53) | HSIL | 100 | 89% male (16M:2F) | Not specified | 100 | Canal | Patients were required to have no more than 3 discrete intra-anal HSIL lesions with positive margins and a maximal diameter of 1 cm. On average, 2.2 HSILs per patient were treated with the IRC at the initial visit. | 12 months | Adverse events  Anal bleeding  Anal discharge  Anal pain  Faecal incontinence  Flatulence  HPV response  Recurrence  Response |
| **JC Singh[20]** | 2009 | USA | Retrospective cohort study (chart review) | **85% Trichloroacetic acid:** first-line treatment consisted of up to four applications of TCA at one-to-two-month intervals. | 54 | Median age 44 HIV +ve (range 33-65), Median age 45 HIV -ve (range 24-71) | 1,2,3 | 48% HIV +ve; 70% HIV negative | 100% male | 100% MSM | 65% HIV +ve (35 participants) | Canal and/or Perianus | Lesions required to be less than one square centimetre in size at the base. Number of participants with: 1 lesion: 13 HIV +ve (37%), 7 HIV -ve (37%); 2 lesions: 8 HIV +ve (23%), 4 HIV -ve (21%); 3 lesions: 7 HIV +ve (20%), 4 HIV -ve (21%); >4 lesions: 7 HIV +ve (20%), 4 HIV -ve (21%). | Not specified | Adverse events  Anal abscess  Anal bleeding  Anal pain  Anal ulceration  Number of treatments to response  Recurrence  Response |
| ***PA Fox[21]** | 2010 | UK | Randomised controlled trial (double-blind, placebo controlled) | **Imiquimod** vs **Placebo:** applied digitally to the canal, three times weekly for 4 months. | 53 (28 Imiquimod; 25 Placebo) | Mean age 41.8 (SD 6.6) Imiquimod; Mean 42.3 (SD 7.4) Placebo | HSIL | 100 | 100% male | 100% MSM | 100 | Canal | Not specified | Mean 36 months | Adherence/Compliance  Progression to ASCC  Recurrence  Response  Retreatment  Tolerability/acceptability of intervention |
| **O Richel[22]** | 2010 | Netherlands | Pilot clinical trial | **5-Fluorouracil:** 1 gram applied intra-anally, at night, Bi-weekly for 16 weeks. | 46 | Median age 46 (range 32-72) | 1,2,3 | 74 | 100% male | 100% MSM | 100 | Canal | 76% (35 patients) had multifocal disease (AIN lesions present in two or more quadrants of the anal circumference). 24% (11 patients) had monofocal disease (a single lesion present in one quadrant of the anal circumference). | 6 months | Adherence/Compliance  Adverse events  Anal bleeding  Anal pain  Anal skin inflammation/irritation (generalised)  Anorectal perforation  Faecal urgency  HPV response  Proctitis  Progression to ASCC  Recurrence  Response  Tolerability/acceptability of intervention |
| **SM Snyder[23]** | 2011 | USA | Retrospective cohort study (chart review) | **Topical 5% 5-Fluorouracil:** pea-sized (approximately 0.25g) amount intra-anally nightly before bed for up to 20 weeks. | 11 | Median age 45 (range 32-67) | 1,2,3 | 60 | 100% male | 100% MSM | 100 | Canal | Diffuse AIN including circumferential disease | Not specified | Adherence/Compliance  Anal fissure  Anal skin inflammation/irritation (generalised)  Herpes simplex virus infection  Response  Tolerability/acceptability of intervention |
| ***SE Weis[24]** | 2012 | USA | Prospective cohort study | **Infrared coagulation (IRC):** single session ablation under LA with additional IRC in participants with ongoing disease at follow up vs **Active monitoring/delayed treatment** | 124 (82 IRC; 22 AM; 20 delayed treatment) | Mean age 39.6 (SD 9.0) | 2,3 | 100 | 79.8% male (99M:25F) | Not specified (52.4% of males Homosexual) | 100 | Canal | Not specified | Mean 1.8 years (SD 1.0) for untreated; 1.3 years (SD 0.8) for treated patients. | Progression to ASCC  Response  Retreatment |
| **DK Marks[25]** | 2012 | USA | Retrospective cohort study (chart review) | **Electrocautery ablation:** Single session ablation of disease) | 232 | Median age 49 HIV +ve (range 27-74); Median age 42 HIV -ve (range 21-70) | HGAIN | 100 | 100% male | 100% MSM | 57% HIV +ve (132 HIV +ve, 100 HIV -ve) | Canal | HIV +ve MSM had significantly more lesions treated at their first ablation than HIV -ve MSM; in total, 375 lesions were treated in HIV +ve MSM versus 226 in HIV -ve MSM. Patients with only 1 HGAIN on first ECA were less likely to have recurrence than patients with 2 or 3 lesions in HIV-positive patients. | Mean 21.1 months (range 6.1-43.9) HIV -ve; 20 months HIV +ve (5.4-46) | Adverse events  Anal bleeding  Anal pain  Anal stenosis  Analgesic use  Progression to ASCC  Recurrence  Response  Superficial local (Anal) infection  Wound healing |
| **EM Van der Snoek[26]** | 2012 | Netherlands | Early phase trial | **Photodynamic therapy**: Treatment directed to entire area; some participants having multiple treatments in the study. | 15 | Median age 46 (IQR 41-54) | 3 | 100 | 100% male | 100% MSM | 100 | Canal and/or Perianus (3 participants perianal; 12 canal) | Only participants with solitary lesions included. No further extent r.e., disease extent. | Variable follow up reported for different outcome. No overall follow-up duration reported | Abdominal pain  Anal bleeding  Anal discharge  Anal itch  Anal pain  Anal skin inflammation/irritation (generalised)  Anal stenosis  Constipation  Fatigue  Faecal urgency  Recurrence  **Response**  Scar upper arm |
| **A Macaya[27]** | 2012 | Spain | Systematic review | Cochrane systematic review of **Imiquimod** for the treatment of anal HSIL inclusive of single study already captured in this review (P Fox 2010). | - | - | - | - | - | - | - | - | - | - | Adverse events  HPV response  Progression to ASCC  Quality of life  Recurrence  **Response**  Tolerability/acceptability of intervention |
| **SA Assoumou[28]** | 2013 | USA | Retrospective cohort study (chart review) | **Electrocautery** **or CO2 laser** **ablation:** single session treatment for disease. | 80 | Median age 42 (range 21-62) | 1,2,3 | 90 | 100% male | 100% MSM | 56% HIV +ve (86 HIV +ve; 67 HIV -ve) | Not specified | Extent of disease measured by the number of anal epithelial surface quadrants involved. 1 quadrant: 26 patients; 2-4 quadrants: 22 patients; Unknown: 1 patient. | Not specified | **Recurrence** |
| **EA Stier[29]** | 2013 | USA | Early phase trial | **1% Cidofovir:** six treatment cycles, one cycle comprising 5 consecutive treatment days followed by 9 non-treatment days. | 33 | Median age 44 (range 24-64) | HGAIN | 100 | 73% male (24M:9F) | Not specified (23 participants reported Homosexual contact) | 100 | Perianal | Participants with perianal AIN covering a surface area of at least 3cm2 included. Median baseline total lesion size was 6.6cm2 (SD 5.4, range 3.0-21.3). | 6 weeks | Adverse events  Anaemia  Anal abscess  Anal bleeding  Anal pain  Anal skin inflammation/irritation (generalised)  Anal ulceration  Constipation  Diarrhoea  Herpes simplex virus infection  HPV response  MRSA infection  Nausea/vomiting  Progression to ASCC  Proteinuria  Respiratory tract infection  Response  Syphilis infection  Vaginal infection  White cell count |
| **G Sirera[30]** | 2013 | Spain | Retrospective cohort study (prospectively collected data) | **Infrared coagulation:** single session ablation under LA with further treatment for recurrence. | 69 | Median age 43 (range 22-58) | 2,3 | 100 | 80% male (59M:14F) | 74% MSM | 100 | Canal | Number of lesions: 68 patients (99%) presented with only one confirmed lesion; 1 patient presented with two confirmed lesions. | 25 | Adverse events  Anal bleeding  Anal pain  Anal stenosis  Analgesic use  HPV response  Progression to ASCC  Recurrence  **Response**  Superficial local (Anal) infection  Wound healing |
| ***O Richel[31]** | 2013 | Netherlands | Randomised controlled trial | **Electrocautery ablation**: single session to all visible lesions every 4 weeks up to 5 sessions) vs **5% Imiquimod:** 6.25mg nocte, three nights/week for 16 weeks vs **2% 5-FU:** 1 gram OD for canal lesions, BD for perianal lesions, twice weekly for 16 weeks. | 148 (46 ECA; 54 Imiquimod; 48 5-FU) | Median age 47 ECA (IQR 42-55); 45 Imiquimod (IQR 41-51): 47 5-FU (IQR 40-54) | 1,2,3 | 54% ECA (25/46); 57% Imiquimod (31/54); 60% 5-FU (29/48) | 100% male | 100% MSM | 100 | Canal and/or Perianus (Canal: ECA 43/46 (93%); Imiquimod 50/54 (93%), 5-FU 47/48 (98%); Perianus: ECA 7/46 (15%); Imiquimod 11/54 (20%), 5-FU 7/48 (15%). | Multifocal AIN (n/N (%)): Electrocautery: 21/46 (46%); Imiquimod: 32/54 (59%); Fluorouracil: 23/48 (48%). | Up to 72 weeks post treatment | Adverse events  Anal bleeding  Anal itch  Anal pain  Anorectal perforation  Diarrhoea  Fatigue  Faecal incontinence  Faecal urgency  Flatulence  Flu-like symptoms  Musculoskeletal injury  Myocardial infarction  Prostatitis  **Recurrence**  **Response**  Slimy stool  Strabismus  Syphilis infection  Tolerability/acceptability of intervention  Treatment related death |
| **RD Cranston[32]** | 2014 | USA | Retrospective cohort study (chart review) | **80% Trichloroacetic acid:** single session for all disease. | 72 | Median age 48 (range, 25-70) | 2,3 | 100 | 100% male | Not specified (but reported predominantly white MSM) | 100 | Canal | Lesion(s) that did not extend individually beyond 25% or collectively beyond 50% of the anal circumference were treated with TCA. A total of 98 HSILs in 72 patients, including 21 with 2 or 3 lesions were identified. | Not specified | Anal pain  Progression to ASCC  Recurrence  Response |
| **AG Smulian[33]** | 2014 | USA | Early phase trial (Phase 1 'dose-ranging') | **Radiofrequency ablation:** participants subjected to increasing RF pulses (phase 1), increased treatment area (phase2)). | 19 (13 phase 1; 6 phase 2) | Not specified | HSIL | 100 | Not specified | Not specified | 100 | Canal | Not specified | At least 18 months | Anal bleeding  Anal discharge  Anal pain  Anal stenosis  Change in stool frequency  Daily activities  Diarrhoea  Faecal incontinence  Non-stool anal symptoms  Tolerability/acceptability of intervention  Wound healing |
| **SE Goldstone[34]** | 2014 | USA | Retrospective cohort study (chart review) | **CO2 Laser ablation** (disease involving more than 50% of the anal canal underwent CO2 laser in the operating room)**;** For all other disease, in office, **Infrared coagulation (IRC)** (Pre 2006) **or Electrocautery ablation** (post 2006): Single stage ablation in all instances | 727 | Median age 44.3 (range 17.4-70.6) HIV +ve; 39.4 HIV -ve (range 211-75) | HSIL | 100 | 100% male | 100% MSM | 63% HIV +ve (456 HIV +ve) | Canal | Any extent of disease | Median 2.23 years (range 0.2-13.2 years) | **Recurrence**  **Progression to ASCC** |
| **EM Van der Snoek[35]** | 2015 | Netherlands | Prospective cohort study | **5% Imiquimod cream:** 6.25mg applied 5 consecutive nights/week for 16 weeks + further 16 weeks if no response. | 44 | Not specified | HSIL | 100 | 100% male | 100% MSM | 100 | Canal and/or Perianus (Canal 37/44 (84.1%); Perianal 7/44 (15.9%) | Not specified | 12 months | Adherence/Compliance  Adverse events  Anal pain  Fatigue  Flu-like symptoms  Mood change  Recurrence  **Response**  Tolerability/acceptability of intervention |
| **A Johnstone[36]** | 2015 | USA | Retrospective cohort study (chart review) | **Electrocautery ablation** (after 2006); **Laser ablation** or **infrared coagulation (IRC)** (before 2006): single session ablation for disease or on rare occasions staged ablation for circumferential disease. | 81 | Median age 44.7 HIV +ve (range 28.1-66.6); 42.8 HIV -ve (range 27.4-68.1) | HSIL | 100 | 100% male | 100% MSM | 86% HIV +ve (70 HIV +ve; 11 HIV -ve) | Perianal | Number of concurrent perianal HSILs at first treatment for both HIV +ve and HIV -ve patients was 1.00 (maximum 9 for HIV +ve and 3 for HIV -ve) | Median 4.62 years (HIV +ve); 3.53 years (HIV -ve) | Anal stenosis  Faecal incontinence  Progression to ASCC  **Recurrence**  Stoma formation  Wound healing |
| **E Sendagorta[37]** | 2016 | Spain | Pilot clinical trial | **1% Cidofovir:** 2g applied intra-anally three times per week for 4 weeks (12 applications). | 17 | Median age 36 (IQR 28-41) | 2,3 | 100 | 100% male | 100% MSM | 100 | Canal | 15 patients (94%) had multifocal involvement. Number of quadrants affected per participant ranged 1-4. | Up to 24 weeks | Adherence/Compliance  Adverse events  Anal bleeding  Anal pain  Diarrhoea  Flatulence  HPV response  Recurrence  **Response** |
| **ML Siegenbeek van Heukelom[38]**  ******** | 2016 | Netherlands | N/A | Study reporting QoL/sexual functioning outcomes from RCT (O Richel 2013). | - | - | - | - | - | - | - | - | - | - | Anxiety  Daily activities  Discomfort/pain with/following intercourse (anal)  Enjoyment of sex  Mobility  Return to sexual activity (anal)  Sex satisfaction  **Sexual functioning**  Unspecified pain |
| ***J Burgos[39]** | 2016 | Spain | Retrospective cohort study (chart review) | **Electrocautery ablation:** every 4-6 weeks for a minimum of two sessions or until visual inspection revealed lesion regression after a maximum of four sessions, all visible lesions being treated in the same session if the extension was less than a quarter circumference. | 83 | Median age 41.2 (IQR 34.1-45.9) | HGAIN | 100 | 100% male | 100% MSM | 100 | Canal | Not specified | Mean 12.2 months (IQR 2-21.3) | Adherence/Compliance  Adverse events  Anal bleeding  Anal pain  Anal stenosis  Analgesic use  Number of treatments to response  Progression to ASCC  Recurrence  **Response**  Superficial local (Anal) infection  Tolerability/acceptability of intervention  Wound healing |
| **WG Willeford[40]** | 2016 | USA | Retrospective cohort study (chart review) | Topical therapy (**5-FU** or **imiquimod** (5%) creams: applied intra-anally for up to 16 weeks or **IRC:** topical for more diffuse disease, IRC for more limited disease. | 49 (36 IRC; 13 Topical) | Median age 42 (range 18-75) | HSIL | 100 | 100% male | Not specified | 100 | Canal | Topicals used for diffuse high-grade AIN, whereas IRC used for more limited disease. No further specification. | Not specified | **Response** |
| **N Willems[41]** | 2017 | Belgium | Retrospective cohort study (chart review) | One or a combination of **ECA**, **IRC**, **surgical treatment** or **5% imiquimod:** choice of treatment depended on the size, number, location and grade of lesions. Other factors that were considered before choosing the treatment were the duration of treatment, the expected treatment-related discomfort, the likelihood that the patient would adhere to treatment, and patient preference. | 73 | Median age 41 (IQR 34-48) | 1,2,3 | 62 | 100% male | 100% MSM | 100 | Canal and/or Perianus | Disease considered extensive if involving 4 quadrants. No further numerical breakdowns. | Median 9.7 months (IQR 2.1-16.8) | Ability to work  Anal bleeding  Anal pain  Anal skin inflammation/irritation (generalised)  Anal stenosis  Faecal incontinence  Progression to ASCC  Recurrence  Response  Superficial local (Anal) infection  Tolerability/acceptability of intervention |
| **RN Goldstone[42]** | 2017 | USA | Early phase trial | **Radiofrequency ablation:** single session hemi-circumferential ablation. | 21 | Mean age 45 (range 32-65) | HSIL only | 100 | 85.7% male (18M:3F) | Not specified | 0 | Canal | Participants with biopsy-proven HSIL (index lesions) involving ≤50 % circumference of the anal squamo-columnar junction (occupying no more than two contiguous quadrants). Mean of 1.7 lesions (range 1-4) per participant were treated. Six (29%) participants had HSIL in two contiguous quadrants. | 12 months | Adverse events  Anal bleeding  Anal discharge  Anal fissure  Anal pain  Anal stenosis  Analgesic use  Appendicitis  Disease free survival  Faecal incontinence  Fever  Hypothyroidism  Intervention for bleeding  Low testosterone  Musculoskeletal injury  Narcotic pain medication addiction  Recurrence  **Response**  Sleep apnoea  Tolerability/acceptability of intervention |
| **RN Goldstone[43]** | 2017 | USA | Pilot clinical trial | **Radiofrequency ablation:** single session circumferential ablation + repeat sessions for recurrence. | 10 | Median age 52 (range 29-70) | aHSIL only | 100 | 100% male | Not specified | 90% HIV +ve (9 HIV +ve; 1 HIV -ve) | Canal | At baseline, participants had a median of 2 (2-8) HSILs. Quadrants with HSIL per participant was 2 (range 1-4). Proximal boundary of canal defined as 3cm proximal to the dentate line. | 12 months | **Adverse events**  Anal bleeding  Anal pain  Anal stenosis  Analgesic use  Asthma  Disease free survival  Ear fluid  Faecal incontinence  Haemorrhoid flare  Quality of life  Recurrence  Response  Return to sexual activity (anal)  Wound healing |
| **RD Cranston[44]** | 2018 | USA | Pilot clinical trial | **5% Imiquimod:** Intra-anally, nightly, three times/week for 9 weeks. | 10 | Median age 46 (IQR 12) | HSIL | 100 | 100% male | 100% MSM | 100 | Canal | The number of HSILs treated was 20 across 9 participants. No participant level data specified. | Not specified | Adherence/Compliance  Adverse events  Anal fissure  Anal skin inflammation/irritation (generalised)  Cytokine gene expression  Fatigue  Fever  Haemorrhoid flare  HIV response  HPV response  Progression to ASCC  Response  Tolerability/acceptability of intervention |
| **ML Siegenbeek van Heukelom [45]** | 2018 | Netherlands | Retrospective cohort study (chart review) | **Cryotherapy:** Multiple treatment sessions with treatment to all lesions per session. Sessions 4-6 weeks apart for a maximum of 5 sessions. | 64 | Median age 48 (IQR 42-56) | HSIL | 100 | 100% male | 100% MSM | 100 | Canal and/or Perianus (1/64 (2%) Perianal; 52/64 (81%) Canal; 11/64 (17%) Both) | Number of quadrants containing HSIL disease: One quadrant: 45/63 (71%); Two quadrants: 12/63 (19%); Three quadrants: 5/63 (8%); Four quadrants: 1/63 (2%). | Up to 18 months | Adverse events  Anal bleeding  Anal fissure  Anal pain  Anxiety  Constipation  Diarrhoea  Erectile function  Faecal incontinence  Haemorrhoid flare  Intervention for adverse effects  Progression to ASCC  Recurrence  Response  Stress  Tolerability/acceptability of intervention |
| **A de Pokomandy[46]** | 2018 | Canada | Pilot clinical trial | **Argon plasma coagulation:** ablation to all disease in single session under LA with additional treatments for persistent/recurrent disease up to a maximum of four, at least 1 month apart. | 20 | Median age 49 (range 37-59 | HSIL | 100 | 100% male | 100% MSM | 100 | Canal | Surface of anal canal affected by prevalent anal HSIL: 0-25%: 12 (60%); 26-50%: 7 (35%); 51-75%: 1 (5%). | 2 years | Adverse events  Anal bleeding  Anal fissure  Anal pain  Anal ulceration  Analgesic use  Anxiety  Arthralgia  Bronchitis  Dental abscess  Epididymitis  Fatigue  Faecal incontinence  Faecal urgency  Fungal infection  Gastrointestinal tract infection  Headache  Herpes simplex virus infection  HPV response  Musculoskeletal injury  Nausea/vomiting  Procedural blood loss  Pyelonephritis post-pyelograph  Recurrence  Renal toxicity  **Response**  Retreatment  Thrombophlebitis  Unspecified infection  Urinary incontinence |
| **J Burgos[47]** | 2018 | Spain | Retrospective cohort study (unspecified data collection) | **Electrocautery ablation:** sessions performed every 4–6 weeks for a minimum of 2 sessions or until visual inspection revealed lesion regression up to a maximum of 4 sessions vs **Topical 85% TCA ablation:** TCA ablation repeated every 4–6 weeks for two-to-four sessions. | 170 | Median age 43.4 (IQR 36.8-49.6) | HSIL | 100 | 100% male | 100% MSM | 100 | Canal | HSIL affecting more than 2 octants of the anal circumference was noted in 52.1% of cases; Multifocal lesions were present in 30.7% of cases; Median No. of octants affected by HSIL 3.0 (IQR 2.0-4.0) for ECA and 2.0 (IQR 2.0-3.0) for TCA, with a p-value of 0.068; Large HSIL (referring to affecting more than 2 octants) was 56.6% for ECA and 37.5% for TCA (P=0.019). | 19.8 months (IQR;12.8–28.9) for ECA group; 4.8 months (IQR; 3.5–9.6) for TCA group | Adverse events  Anal bleeding  Anal itch  Anal pain  Progression to ASCC  Recurrence  **Response**  Tolerability/acceptability of intervention |
| ***SE Goldstone[48]** | 2019 | USA | Randomised controlled trial | **Infrared Coagulation**: All lesions treated in a single session under LA, up to 3 sessions depending on response vs **Active monitoring** | 120 (60 IRC; 60 AM) | Mean age 49 (range 25-78) IRC; 50.5 (27-67) AM | 2,3 | 100 | 90% male (54M:6F) IRC; 97% male (58M:2F) AM | Not specified | 100 | Canal | Participants with 1-3 canal lesions (15mm in maximum diameter) were enrolled. Number of lesions per participant for IRC: 1 lesion: 41 (68%); 2 lesions: 17 (28%); 3 lesions: 2 (3%). For AM: 1 lesion: 32 (53%); 2 lesions: 19 (32%); 3 lesions: 9 (15%). | Up to 24 months | Adverse events  Anal bleeding  Anal pain  Anxiety  Aortic valve disease  Basal cell carcinoma  Coronary artery disease  Disease free survival  Gastrointestinal tract infection  Lymphoma  Musculoskeletal injury  Pericardial effusion  Progression to ASCC  Recurrence  **Response**  Retinal vascular disorders  Retreatment  Thromboembolic event |
| **J Corral[49]** | 2019 | Spain | Systematic review | Systematic review of **Infrared Coagulation** comprising 6 studies included in this review | - | - | - | - | - | - | - | - | - | - | Adverse events  Anal bleeding  Anal discharge  Anal pain  Anal stenosis  Faecal incontinence  Flatulence  **Progression to ASCC**  Recurrence  Superficial local (Anal) infection |
| **MM Gaisa[50]** | 2020 | USA | Retrospective cohort study (chart review) | **Electrocautery ablation:** All lesions ablated in single visit | 330 | Median age 45.5 (IQR 35-51) | HSIL | 100 | 88% male | 88% MSM | 100 | Canal | 51% of patients were found to have a solitary index HSIL; 49% had multiple index HSILs (range 2-6). | Median 12.2 months (IQR 6.3-20.9) | Progression to ASCC  Recurrence |
| **EA Stier[51]** | 2020 | USA | Retrospective cohort study (chart review) | One or more of: **Ablation, Excision, Hyfrecation, IRC, Topicals (not specified).** | 45 (operating room (OR) with ablation and/or excision (53%); 35% were treated with office ablative procedures (hyfrecator) (20%); IRC (15%); 10% topical therapies) | Median age 46 (range 35-66) | 2,3 | 100 | 100% female | N/A | 100 | Not specified | Not specified | Up to 3 years | Progression to ASCC  Recurrence |
| **D Brogden[52]** | 2021 | UK | Systematic review | Systematic review of **various treatments** for AIN comprising 30 studies included in this review | - | - | - | - | - | - | - | - | - | - | Anal bleeding  Anal pain  Anal skin inflammation/irritation (generalised)  Anal stenosis  Progression to ASCC  Recurrence  Response  Sexual functioning  Tolerability/acceptability of intervention |
| **J Corral[53]** | 2021 | Spain | Retrospective cohort study (prospectively collected data) | **Infrared Coagulation:** single treatment to all known lesions | 81 | Median age 44 (range 24-77) | 2,3 | 100 | 80% male (65M:16F) | 92% MSM (60-65) | 100 | Canal | At baseline, 101 anal lesions were identified in 81 patients. 30% (19/65) of the men had >1 lesion; all the women had only one lesion. | Median 55 months (range 33-71) | Adverse events  Anal pain  Recurrence |
| **O Vergara-Fernandez[54]** | 2021 | Mexico | Retrospective cohort study (prospectively collected data) | **Radiofrequency ablation:** Single session treating all disease with subsequent session for ongoing disease and if remaining HSIL then targeted ECA. | 12 | Mean age 38.6 (range 26-57) | HSIL | 100 | 58% male (7M:5F) | Not specified | 50 | Canal | A mean of 2.1 anal HSILs (range 1-5) per patient were treated. | Mean follow-up 18 months | Anal bleeding  Anal canal structure  Anal discharge  Anal fissure  Anal pain  Analgesic use  Anorectal function  Anxious symptoms  Daily activities  Depressive symptoms  Desire for sex  Emotional wellbeing  Erectile function  General health  Mobility  Orgasm  Physical functioning  Recurrence  **Response**  Retreatment  Sex satisfaction  Sexual arousal  Sexual functioning  Social activities  Social functioning  Symptom response  Unspecified infection  Unspecified pain  Wound healing |
| **I Fuertes[55]** | 2021 | Spain | Retrospective cohort study (prospectively collected data) | **Electrocautery:** all lesions treated in a single session up to half the anal circumference, with subsequent sessions at 3-5 month intervals until clearance. | 91 | Mean 42 (IQR 36-46) | HSIL | 100 | 100% male | 100% MSM | 100 | Canal | 128 HSILs in 91 men were included in this study. Number of anal HSIL/participant was evaluated: 1 (52%), 2 (27%), 3 or more (22%). | Mean 760 days (IQR 446-1197) | Adverse events  Anal bleeding  Anal pain  Recurrence  Response  Unspecified infection |
| ***C Hidalgo-Tenorio[56]** | 2021 | Spain | Retrospective cohort study (prospectively collected data) | **Electrosurgical mucosal resection** vs **5% Imiquimod**: 3 times/week for 16 weeks. If imiquimod failed, patients were offered extension of the course up to 18 weeks, a new 16-week cycle of imiquimod, or surgery. | 79 (Imiquimod as first option (n =32); Surgery as first option (n=47)) | Mean age: Imiquimod 35.3 (+/- 11.48); Surgery 31.3 (+/- 8.3) | HSIL | 100 | 100% male | 100% MSM | 100 | Canal | The median number of affected quadrants was 1 (IQR: 1-2) for the imiquimod group. No specific detail on lesion size or number for the surgery group provided. | Mucosectomy: median 60 months (IQR: 46–73 months); Imiquimod (mean) 48 months (IQR: 35–57 months) | Adverse events  Anal bleeding  Anal itch  Anal pain  Anal stenosis  Faecal incontinence  HPV response  Progression to ASCC  Recurrence  Resection margin involvement  Response  Retreatment  Tolerability/acceptability of intervention |
| ***JM Palefsky[57]** | 2022 | USA | Randomised controlled trial | Treatment vs Active monitoring: Treatments included ablative procedures (**infrared coagulation, electrocautery,** and **laser**), ablation or excision under anaesthesia, and topical treatments (**imiquimod** and **fluorouracil**). Treatment included office-based ablative procedures, ablation or excision under anaesthesia, or the administration of topical fluorouracil or imiquimod. Participants treated until HSIL completely resolved. | 4459 (2237 Treatment; 2222 AM) | Median age 51 (IQR 44-57) | HSIL | 100 | Treatment:1793 (80.5%) M: 346 (15.5%) F: 85 (3.1%) Trans: Other 3 (0.2%) AM: 1782 (80.5%) M: 365 (15.5%) F: 68 (3.1%) Trans: Other 4 (0.2%). | Treatment: 1716 (77.1%) MSM; AM: 1717 (77.4%) MSM. | 100 | Canal and/or perianus | Lesion size at randomization was a stratification factor (≤50% vs. >50% of the anal canal or perianal region). >50% of anal canal or perianal region: 285 (12.8%) in treatment group, 282 (12.7%) in AM group. | Treatment 25.3 (IQR 11.7-42); AM 27.2 (IQR 12-42.1) | Adverse events  Anal abscess  Anal pain  Anal ulceration  Death  **Progression to ASCC**  Superficial local (Anal) infection  Tolerability/acceptability of intervention  Treatment related death |
| **I Fuertes[58]** | 2022 | Spain | Pilot clinical trial | **CO2 Laser ablation:** all disease treated in a single session with subsequent sessions for persistent/recurrent disease. | 52 | Mean age 45.1 (range 23.3-64.8) | 2 (P16), 3 | 100 | 96% male (50M:2F) | Not specified | 100 | Canal | Number of HSIL per patient: 1 HSIL: 37 patients (71.2%); 2 HSILs: 10 patients (19.2%); 3 HSILs: 5 patients (9.6%). | Median 133.5 days (range 35-301 days) | Adverse events  Anal bleeding  Anal pain  Recurrence  Response |
| **CG Martínez[59]** | 2023 | Spain | Retrospective cohort study (unspecified data collection) | **80% Trichloroacetic acid:** two applications (to entire anal canal mucosa) of TCA 30 days apart with 2 further applications for persisting disease. | 115 | Mean age 42.5 (range 19-65) | 1,2,3 | 43 | 92% male (106M:9F) | 50% MSM | 90 | Canal | Not specified | Up to 1 year | Recurrence  Response |
| **J Burgos[60]** | 2023 | Spain | Prospective cohort study | **1% Cidofovir:** applied three times weekly for 8 weeks. | 23 | Median age 48 (IQR 39.9-53.3) | HSIL | 100 | Men and transgender people who have sex with men with HIV. The exact proportion of men vs. transgender people is not specified. | Not specified | 100 | Canal | HSIL for at least 50% of the anal circumference was noted in 39% of cases. Multifocal lesions were present in 30.4%. The median number of affected octants was 3 (IQR 2-4). | Median 30.3 months (IQR 6.9-41.8) | Adherence/Compliance  Adverse events  Anal itch  Anal pain  Anal skin inflammation/irritation (generalised)  HPV response  Recurrence  Response  Tolerability/acceptability of intervention |
| **KCM Gosens[61]** | 2023 | Netherlands | Early phase (dose finding) trial (Phase 1 to 2) | **Therapeutic vaccine SLP-HPV-01:** four different dosage schedules (1-5-10; 5-10-20; 10-20-40; and 40-40-40-40 mg of SLP- HPV-01 administered intradermally with a 3-week interval. | 40 | Median age 53.5 (IQR 46.3-58.5) | HGAIN | 100 | 100% male | 100% MSM | 100 | Canal | Not specified | Up to 18 months | Abdominal pain  Anaemia  Anal itch  Arthralgia  Bone pain  Concentration problems  Coughing  Depression  Diarrhoea  Dizziness  Dry mouth  Dry skin  Dyspnoea  Fatigue  Fever  Flu-like symptoms  Foul breath  Hair loss  Headache  HPV immune response  Injection site pain/reaction  Liver function  Loss of appetite  Mood change  Muscular pain  Musculoskeletal injury  Nausea/vomiting  Palmar Erythema  Platelets  Rash  Respiratory tract infection  **Response**  Sleep disturbance  **Tolerability/acceptability of intervention**  Vasovagal collapse  Weight loss  White cell count |
| **SH Fang[62]** | 2023 | USA | Early phase trial (Phase 1) | **Artesunate suppositories:** administered intra-anally (doses: 200mg, 400mg, 600mg). The lowest dose level was two 5-day cycles of artesunate, followed by three five-day cycles of artesunate. In the absence of toxicity, patients were enrolled at the next highest dose in the same manner. | 19 | Median age 49 (range 26-60) | AIN 2, 3, HSIL | 100 | 63% male (12M:7F) | 100% MSM | 37 | Canal | Lesion size. At screening HRA, all study participants had residual anal HSIL as documented by HRA, which ranged from minimal disease (1 or 2 small lesions, < 5 mm in size) to extensive intra-anal and perianal disease. Partial regression is defined as: (1) clearance of intra- anal HSIL, but not perianal HSIL in a patient who had both or (2) >50% reduction of the area of HSIL lesions compared to the area of anal HSIL seen on screening HRA | Up to 40 weeks | Abdominal pain  Anal bleeding  Anal itch  Anal pain  Anal skin inflammation/irritation (generalised)  Anxiety  Bruising  Constipation  Diabetic ketoacidosis  Diarrhoea  Dizziness  Dry mouth  Esophageal infection  Fatigue  Fever  Flatulence  Headache  HPV response  Low back pain  Muscular pain  Nausea/vomiting  Paraesthesia  Rash  Response  Sore throat  Toenail fungus  **Tolerability/acceptability of intervention**  Unspecified pain  Vertigo |
| **F Singhartinger[63]** | 2024 | Austria | Retrospective cohort study (prospectively collected data) | **Endoscopic submucosal dissection** | 15 | Median age 51.7 (range 23-78) | AIN 3 | 100 | 40% male (6M:9F) | Not specified | Not specified | Canal | Not specified | Median 11.7 months (range 2-23 months) | Adverse events  Anal bleeding  Anal stenosis  Duration of intervention  Intervention for bleeding  Progression to ASCC  Recurrence  Resection margin involvement  Retreatment |
| ***P Borch-Johnsen[64]** | 2024 | Sweden | Retrospective cohort study (chart review) | **Endoscopic underwater resection** or **Surgical excision:** In the case of multifocal ASIL covering almost the entire circumference, resection of 40–50% was carried out during the first session. | 80 (37 Endoscopy, 43 surgery) | Mean age (SD): Endoscopy 48.7 +/- 20.7, Surgery 48.8 +/- 15.7 | AIN 1,2,3 | Endoscopy 46%, Surgery 37%) | Endoscopy 62% (23M), Surgery 65% (29M)) | Not specified | Not specified | Endoscopy, Canal; Surgery, Canal and Perianus | Unifocal and multifocal lesions (2,3,4,5 or more lesions) | Not specified | Anal stenosis  Faecal incontinence  Intervention for bleeding  Number of treatments to response  Procedural blood loss  Recurrence  Rehospitalisation  Tolerability/acceptability of intervention |
| ***J Burgos[65]** | 2024 | Spain | Prospective cohort study | **85% Trichloroacetic acid:** repeated every 6–8 weeks for a minimum of two sessions and a maximum of four sessions. **Electrocautery:** performed every 6 weeks for a minimum of two sessions and a maximum of four sessions. | 487 (227 ECA, 260 TCA) | Mean age (SD): ECA 43.8 +/- 10.3, TCA 44.7 +/- 9.8 | HSIL | 100 | 100 | 100% MSM | 100 | Canal | At the moment of the treatment, HSIL involving more than half of the anal circumference was noted in 20.5% of cases and multifocal lesions were noted in 28.5% determined by number of octants affected. Multifocal HSIL present in 33% ECA group and 26.45 TCA group. | Median 24.1 months (IQR 12.6–41.8 months) | Adverse events  Anal bleeding  Anal itch  Anal pain  Faecal urgency  HPV response  Recurrence  Response  Retreatment  Tolerability/acceptability of intervention |
| **H Kitamura[66]** | 2025 | Japan | Pilot clinical trial | **Electrocautery:** Single session. In cases with extensive lesions, staged ablations were permitted at the discretion of the physician. | 20 | Mean age 45 (range 27-68) | AIN 2,3 | 100 | 100% male | 100% MSM | 90 | Not specified | Based on an extensive assessment of sextant biopsies, the median area of HSIL involvement was 58.3 % (range 16.7%–100.0 %). | Up to 6 months | Anal bleeding  Anal pain  Intervention for bleeding  Recurrence  **Response** |
| ***J Burgos[67]** | 2025 | Spain | Randomised Controlled Trial | **Electrocautery:** Electrocautery sessions were performed every 8 weeks for a total of 3 sessions, **1% Cidofovir:** applied intra-anally 3 times a week for a total of 8 weeks (total dose of 48 g at 1%), **10% Sinecatechins:** applied intra-anally at a dose of 1g 3 times a week for a total of 8 weeks (total dose of 24 g at 10%). | 108 | Mean age (SD): ECA 44 (12.1), Cidofovir 42.4 (7.8), Sinecatechins 43.4 (9.4) | HSIL | 100 | 100% male/transwomen | 100% MSM | 100 | Canal | HSIL ≥50% of anal circumference (ECA 18.4%, Cidofovir 16.7 %, Sinecatechins 17.5%).  Multifocal current HSIL (ECA 36.8%, Cidofovir 26.7 %, Sinecatechins 22.5%). | Up to 2 years | Adverse events  Anal bleeding  Anal itch  Anal pain  Anal skin inflammation/irritation (generalised)  HPV response  Progression to ASCC  Recurrence  **Response**  Tolerability/acceptability of intervention |
| **TM Atkinson[68]******** | 2025 | USA | N/A | Study reporting QoL outcomes from a subset of participants from J Palefsky 2022 RCT. Outcomes reported are those of the QoL assessment tool developed through the mixed methods study (Burkhalter 2018) | 124 (70 Treatment arm, 54 Active monitoring) | Mean 52.6 (SD 10.3) | HSIL | 100 | 81.5 % male | Not specified | 100 | Cana, and Peroanus | N/A | At randomisation; 2-7 days post treatment; 28 days post treatment | Ability to work  Anal bleeding  Anal discharge  Anal itch  Anal pain  Anxious symptoms  Concentration problems  Constipation  Daily activities  Depressive symptoms  Desire for sex  Enjoyment of sex  Faecal urgency  Intimate relationships  Mobility  Physical functioning  Psychological symptoms  Sitting  Social activities  Symptom response  Unspecified pain  Worried about condition getting worse |
| **N Gallio[69]** | 2025 | Italy | Systematics review | Systematic review of **Imiquimod** for AIN comprising 5 studies included in this review |  |  |  |  |  |  |  |  |  |  | Adverse events  Progression to ASCC  Recurrence  Response  Tolerability/acceptability of intervention |

Table S3. List of verbatim outcomes and ‘standardised outcome terms’ by core area and outcome domain.

| Core area (n=5) | Outcome domain (OD) (n=31) | Number (%) of studies OD reported in | Standardised outcome term (SOT) (n=170) | Number (%) of studies SOT reported in (%) | Verbatim outcome term (VOT) (n=568) | Number of studies VOT reported in |
| --- | --- | --- | --- | --- | --- | --- |
| Physiological/clinical | Blood and lymphatic | 3 (4) | Anaemia | 2 (3) | Anaemia | 2 |
|  |  |  | Blood cell count | 1 (1) | Complete blood cell count | 1 |
|  |  |  | Coagulation | 1 (1) | Partial thromboplastin time | 1 |
|  |  |  |  |  | Prothrombin time | 1 |
|  |  |  | Platelets | 2 (3) | Platelet count | 1 |
|  |  |  |  |  | Thrombopenia | 1 |
|  |  |  | White cell count | 2 (3) | Leucocytosis | 1 |
|  |  |  |  |  | Leukopenia | 1 |
|  |  |  |  |  | Neutrophil count | 1 |
|  | Cardiac | 5 (7) | Aortic valve disease | 1 (1) | Aortic valve disease | 1 |
|  |  |  | Blood pressure | 1 (1) | Systolic blood pressure | 1 |
|  |  |  | Coronary artery disease | 1 (1) | Other cardiac disorder (coronary artery disease) | 1 |
|  |  |  | Myocardial infarction | 2 (3) | Myocardial infarction | 2 |
|  |  |  | Pericardial effusion | 1 (1) | Pericardial effusion | 1 |
|  |  |  | Thromboembolic event | 1 (1) | Thromboembolic event | 1 |
|  |  |  | Vasovagal collapse | 1 (1) | Vasovagal collapse | 1 |
|  | Ear and labyrinth | 2 (3) | Ear fluid | 1 (1) | Ear fluid | 1 |
|  |  |  | Vertigo | 1 (1) | Vertigo | 1 |
|  | Endocrine | 3 (4) | Blood sugar | 1 (1) | Blood sugar | 1 |
|  |  |  | Diabetic ketoacidosis | 1 (1) | Diabetic ketoacidosis | 1 |
|  |  |  | Hypothyroidism | 1 (1) | Hypothyroidism | 1 |
|  |  |  | Low testosterone | 1 (1) | Low testosterone | 1 |
|  | Eye | 2 (3) | Retinal vascular disorders | 1 (1) | Retinal vascular disorders | 1 |
|  |  |  | Strabismus | 1 (1) | Strabismus | 1 |
|  | Gastrointestinal | 54 (81) | Abdominal pain | 3 (4) | Abdominal pain | 3 |
|  |  |  | Anal abscess | 4 (6) | Abscess | 1 |
|  |  |  |  |  | Anal abscess due to electrocautery | 1 |
|  |  |  |  |  | Perianal abscess | 1 |
|  |  |  |  |  | Perirectal abscess | 1 |
|  |  |  | Anal bleeding | 35 (52) | Acute anal bleeding | 1 |
|  |  |  |  |  | Anal bleeding | 5 |
|  |  |  |  |  | Anal bleeding without a bowel movement | 1 |
|  |  |  |  |  | Anal/rectal bleeding | 1 |
|  |  |  |  |  | Anal haemorrhage | 1 |
|  |  |  |  |  | Bleeding | 5 |
|  |  |  |  |  | Bleeding associated with defecation | 2 |
|  |  |  |  |  | Bleeding from the anus | 1 |
|  |  |  |  |  | Bleeding from the perianus | 1 |
|  |  |  |  |  | Bleeding not requiring transfusion | 1 |
|  |  |  |  |  | Bleeding requiring reoperation | 1 |
|  |  |  |  |  | Bleeding with defecation | 1 |
|  |  |  |  |  | Bleeding with stool | 1 |
|  |  |  |  |  | Bowel movement associated bleeding | 1 |
|  |  |  |  |  | Delayed postoperative bleeding | 1 |
|  |  |  |  |  | Excessive bleeding | 1 |
|  |  |  |  |  | Haemorrhage | 1 |
|  |  |  |  |  | Limited bleeding | 1 |
|  |  |  |  |  | Mild bleeding | 2 |
|  |  |  |  |  | Mild blood loss | 1 |
|  |  |  |  |  | Minor anal bleeding | 1 |
|  |  |  |  |  | Non stool anal bleeding | 1 |
|  |  |  |  |  | Persistent bleeding | 3 |
|  |  |  |  |  | Post procedure haemorrhage | 1 |
|  |  |  |  |  | Postoperative haemorrhage/anal haemorrhage | 1 |
|  |  |  |  |  | Rectal blood loss | 1 |
|  |  |  |  |  | Resolution of bleeding associated defecation | 1 |
|  |  |  |  |  | Self-limited bleeding | 2 |
|  |  |  |  |  | Severe bleeding | 1 |
|  |  |  |  |  | Significant postoperative haemorrhage | 1 |
|  |  |  | Anal canal structure | 1 (1) | Anal canal structure | 1 |
|  |  |  | Anal discharge | 7 (10) | Anal discharge | 1 |
|  |  |  |  |  | Anal discharge (mucous) | 1 |
|  |  |  |  |  | Anal mucous discharge | 2 |
|  |  |  |  |  | Anal pocket with purulent discharge | 1 |
|  |  |  |  |  | Discharge (wetness) in my anal area | 1 |
|  |  |  |  |  | Rectal mucous discharge | 1 |
|  |  |  | Anal fissure | 8 (12) | Anal fissure | 7 |
|  |  |  |  |  | Postoperative fissure | 1 |
|  |  |  | Anal itch | 10 (15) | Anal itching | 2 |
|  |  |  |  |  | Anal pruritis | 1 |
|  |  |  |  |  | Itch | 2 |
|  |  |  |  |  | Itching | 3 |
|  |  |  |  |  | Itching in or around the anus | 1 |
|  |  |  |  |  | Perianal itching | 1 |
|  |  |  |  |  | Perianal pruritis | 1 |
|  |  |  | Anal pain | 40 (60) | Anal burning sensations | 1 |
|  |  |  |  |  | Anal discomfort | 7 |
|  |  |  |  |  | Anal Pain | 12 |
|  |  |  |  |  | Anal pain duration | 1 |
|  |  |  |  |  | Anal pain or tenderness during and/or following treatment | 1 |
|  |  |  |  |  | Anal stinging | 1 |
|  |  |  |  |  | Anal spasm | 1 |
|  |  |  |  |  | Anal/rectal pain | 1 |
|  |  |  |  |  | Burning | 2 |
|  |  |  |  |  | Burning sensation | 1 |
|  |  |  |  |  | Burning sensations in the anal area | 1 |
|  |  |  |  |  | Discomfort passing stool | 1 |
|  |  |  |  |  | Local discomfort | 2 |
|  |  |  |  |  | Local Pain | 1 |
|  |  |  |  |  | Mild local pain | 1 |
|  |  |  |  |  | Mild pain | 1 |
|  |  |  |  |  | Pain | 10 |
|  |  |  |  |  | Pain (Anal) | 1 |
|  |  |  |  |  | Pain (During bowel movements) | 1 |
|  |  |  |  |  | Pain associated with bowel movements | 2 |
|  |  |  |  |  | Pain due to electrocautery | 1 |
|  |  |  |  |  | Pain due to infrared coagulation | 1 |
|  |  |  |  |  | Pain due to treatment under anaesthesia | 1 |
|  |  |  |  |  | Pain during defecation | 1 |
|  |  |  |  |  | Pain post anal intercourse | 1 |
|  |  |  |  |  | Pain requiring anti-analgesics | 1 |
|  |  |  |  |  | Pain uncontrolled by PO analgesia | 1 |
|  |  |  |  |  | Pain when opening bowels | 1 |
|  |  |  |  |  | Pain/discomfort during treatment | 1 |
|  |  |  |  |  | Post-ablative pain | 1 |
|  |  |  |  |  | Post procedure anal pain | 1 |
|  |  |  |  |  | Post procedure pain | 1 |
|  |  |  |  |  | Procedural pain | 1 |
|  |  |  |  |  | Uncontrollable pain | 1 |
|  |  |  |  |  | Uncontrolled post procedure pain | 1 |
|  |  |  | Anal scarring | 1 (1) | Scar formation | 1 |
|  |  |  | Anal sensation | 1 (1) | Anoderm sensation | 1 |
|  |  |  | Anal skin inflammation/irritation | 11 (16) | Anal irritation | 4 |
|  |  |  |  |  | Anal mucositis | 1 |
|  |  |  |  |  | Erythema | 1 |
|  |  |  |  |  | Mild local irritation | 1 |
|  |  |  |  |  | Perianal inflammation | 2 |
|  |  |  |  |  | Perianal irritation | 1 |
|  |  |  |  |  | Perineal inflammation | 1 |
|  |  |  |  |  | Pruritis of the anus/perianus/perineum | 1 |
|  |  |  |  |  | Severe anal irritation | 1 |
|  |  |  |  |  | Swelling of existing perianal skin tags | 1 |
|  |  |  | Anal skin pigment change | 1 (1) | Mild Hypopigmentation | 1 |
|  |  |  | Anal stenosis | 22 (33) | Anal stenosis | 7 |
|  |  |  |  |  | Anal stricture | 9 |
|  |  |  |  |  | Clinically significant stenosis | 1 |
|  |  |  |  |  | Irreversible rectal stricture | 1 |
|  |  |  |  |  | Soft anal stricture | 1 |
|  |  |  |  |  | Stenosis | 1 |
|  |  |  |  |  | Transient anal stenosis | 1 |
|  |  |  |  |  | Worsening anal stenosis | 1 |
|  |  |  | Anal ulceration | 5 (7) | Local ulceration | 1 |
|  |  |  |  |  | Mild erosions | 1 |
|  |  |  |  |  | Severe erosions | 1 |
|  |  |  |  |  | Skin ulceration due to fluorouracil | 1 |
|  |  |  |  |  | Ulcer | 1 |
|  |  |  |  |  | Ulceration of the perianus/perineum | 1 |
|  |  |  | Anoderm thinning | 1 (1) | Minor thinning of anoderm | 1 |
|  |  |  | Anorectal function | 1 (1) | Anorectal function | 1 |
|  |  |  | Anorectal perforation | 2 (3) | Anal/rectal perforation | 1 |
|  |  |  |  |  | Rectal perforation caused corpus alienum | 1 |
|  |  |  | Appendicitis | 1 (1) | Appendicitis | 1 |
|  |  |  | Change in stool frequency | 1 (1) | Change in stool frequency | 1 |
|  |  |  | Constipation | 5 (7) | Constipation | 5 |
|  |  |  | Diarrhoea | 7 (10) | Diarrhoea | 6 |
|  |  |  |  |  | Watery stool | 1 |
|  |  |  | Dry mouth | 2 (3) | Dry mouth | 2 |
|  |  |  | Esophageal infection | 1 (1) | Esophageal infection | 1 |
|  |  |  | Faecal incontinence | 17 (25) | Anal incontinence | 2 |
|  |  |  |  |  | Continence difficulties | 1 |
|  |  |  |  |  | Faecal incontinence | 1 |
|  |  |  |  |  | Faecal soiling requiring use of pads | 4 |
|  |  |  |  |  | Incontinence | 5 |
|  |  |  |  |  | Incontinence to formed stool | 1 |
|  |  |  |  |  | Mild faecal incontinence | 1 |
|  |  |  |  |  | Rectal incontinence | 1 |
|  |  |  |  |  | Temporary faecal incontinence | 1 |
|  |  |  | Faecal urgency | 6 (9) | Minor urge to defecate | 1 |
|  |  |  |  |  | Strong urge to defecate | 2 |
|  |  |  |  |  | Tenesmus | 2 |
|  |  |  |  |  | Urge to defecate | 1 |
|  |  |  |  |  | Urgency for bowel movements | 1 |
|  |  |  | Flatulence | 5 (7) | Flatulence | 5 |
|  |  |  | Foul breath | 1 (1) | Foul breath | 1 |
|  |  |  | Haemorrhoid flare | 3 (4) | Haemorrhoid flare | 1 |
|  |  |  |  |  | Haemorrhoid | 1 |
|  |  |  |  |  | Thrombosed external pile | 1 |
|  |  |  |  |  | Ulcerated Haemorrhoid | 1 |
|  |  |  | Loss of appetite | 1 (1) | Loss of appetite | 1 |
|  |  |  | Nausea/vomiting | 5 (7) | Nausea | 2 |
|  |  |  |  |  | Nausea and vomiting | 1 |
|  |  |  |  |  | Nausea/vomiting | 2 |
|  |  |  |  |  | Vomiting | 2 |
|  |  |  | Non-stool anal symptoms | 1 (1) | Non-stool anal symptoms | 1 |
|  |  |  | Proctitis | 1 (1) | Proctitis | 1 |
|  |  |  | Slimy stool | 1 (1) | Slimy stool | 1 |
|  |  |  | Superficial local (Anal) infection | 12 (18) | Anal Infection requiring oral antibiotic | 1 |
|  |  |  |  |  | Cellulitis at local anaesthetic injection site | 2 |
|  |  |  |  |  | Infection | 1 |
|  |  |  |  |  | Infection necessitating antibiotics | 1 |
|  |  |  |  |  | Infection or abscess due to anal biopsy | 1 |
|  |  |  |  |  | Infection requiring antibiotic therapy | 3 |
|  |  |  |  |  | Local infection | 1 |
|  |  |  |  |  | Local infection requiring antibiotic | 1 |
|  |  |  |  |  | Superficial wound infection | 1 |
|  |  |  | Weight loss | 1 (1) | Weight loss | 1 |
|  | General | 14 (21) | Fatigue | 8 (12) | Fatigue | 7 |
|  |  |  |  |  | Fatigue impacting daily activities | 1 |
|  |  |  |  |  | Malaise | 2 |
|  |  |  |  |  | Tired/Asthenia | 1 |
|  |  |  | Fever | 6 (9) | Chills | 2 |
|  |  |  |  |  | Fever | 5 |
|  |  |  | Flu-like symptoms | 4 (6) | Flu-like symptoms | 2 |
|  |  |  |  |  | Influenza like symptoms | 2 |
|  |  |  | General health | 1 (1) | General health | 1 |
|  |  |  |  |  | In general, would you say your health is: | 1 |
|  |  |  |  |  | I seem to get sick a little easier than other people | 1 |
|  |  |  |  |  | I am as healthy as anybody I know | 1 |
|  |  |  |  |  | I expect my health to get worse | 1 |
|  |  |  |  |  | My health is excellent | 1 |
|  |  |  | Injection site pain/reaction | 3 (4) | Arm pain | 1 |
|  |  |  |  |  | Injection site hypesthesia | 1 |
|  |  |  |  |  | Injection site pain | 1 |
|  |  |  |  |  | Injection site reaction | 1 |
|  |  |  |  |  | Injection-site reaction | 1 |
|  |  |  | Rigors and chills | 1 (1) | Rigors and chills | 1 |
|  |  |  | Sweats | 2 (3) | Diaphoresis | 1 |
|  |  |  |  |  | Sweats | 1 |
|  |  |  | Unspecified (bodily) pain | 3 (4) | Bodily pain | 1 |
|  |  |  |  |  | Bodily pain (amount) | 1 |
|  |  |  |  |  | Bodily pain interfering with normal work (including both work outside the home and housework) | 1 |
|  |  |  |  |  | Pain | 1 |
|  |  |  |  |  | Pain / Discomfort | 1 |
|  | Hepatobiliary | 2 (3) | Liver function | 2 (3) | Alkaline phosphatase activity | 1 |
|  |  |  |  |  | Bilirubin | 1 |
|  |  |  |  |  | Elevated AFP | 1 |
|  |  |  |  |  | Elevated AST/ALT | 1 |
|  |  |  |  |  | Elevated GGT | 1 |
|  |  |  |  |  | Elevated bilirubin | 1 |
|  |  |  |  |  | Serum glutamic-oxaloacetic transaminase | 1 |
|  |  |  |  |  | Serum glutamic-pyruvic transaminase | 1 |
|  |  |  |  |  | Severe transaminase elevation | 1 |
|  | Immune system | 4 (6) | CD4 T cell count response | 1 (1) | CD4 T cell count response | 1 |
|  |  |  | CD8 T cell count response | 1 (1) | CD8 T cell count response | 1 |
|  |  |  | Cytokine gene expression | 1 (1) | Cytokine gene expression | 1 |
|  |  |  | HPV immune response | 2 (3) | HPV PBMC response | 1 |
|  |  |  |  |  | HPV16-specific T cell response | 1 |
|  | Infection and infestation | 23 (34) | Dental abscess | 1 (1) | Dental abscess | 1 |
|  |  |  | Fungal infection | 2 (3) | Groin fungal cutaneous infection | 1 |
|  |  |  |  |  | Toenail fungus | 1 |
|  |  |  | Gastrointestinal tract infection | 2 (3) | Giardiasis | 1 |
|  |  |  |  |  | Salmonella poisoning | 1 |
|  |  |  | Herpes simplex virus infection | 3 (4) | Anal/Perianal Herpes | 1 |
|  |  |  |  |  | HSV | 1 |
|  |  |  |  |  | Perianal herpes simplex | 1 |
|  |  |  |  |  | Shingles | 2 |
|  |  |  | HIV response | 2 (3) | Anal and Rectal HIV-1 RNA and DNA quantification | 1 |
|  |  |  |  |  | HIV viral load response | 1 |
|  |  |  | HPV response | 17 (25) | Anal HPV typing | 1 |
|  |  |  |  |  | Clearance of HPV | 2 |
|  |  |  |  |  | Clearance of oncogenic HPV genotype | 1 |
|  |  |  |  |  | Cumulative HrHPV-DNA load response | 1 |
|  |  |  |  |  | HPV 16 response | 1 |
|  |  |  |  |  | HPV Analysis | 1 |
|  |  |  |  |  | HPV DNA load response | 1 |
|  |  |  |  |  | HPV genotype response | 2 |
|  |  |  |  |  | HPV load | 1 |
|  |  |  |  |  | HPV response | 2 |
|  |  |  |  |  | HPV type | 2 |
|  |  |  |  |  | HPV Type response | 4 |
|  |  |  |  |  | HPV-DNA load | 1 |
|  |  |  |  |  | HrHPV viral load response | 1 |
|  |  |  |  |  | Human papilloma virus (HPV) eradication | 1 |
|  |  |  | MRSA infection | 1 (1) | MRSA (thigh) | 1 |
|  |  |  | Respiratory tract infection | 2 (3) | Lung infection | 1 |
|  |  |  |  |  | Pharyngitis | 1 |
|  |  |  |  |  | Upper respiratory infection | 1 |
|  |  |  | Syphilis infection | 2 (3) | Syphilis | 2 |
|  |  |  | Unspecified infection | 3 (4) | Infections | 2 |
|  |  |  |  |  | Non-surgical site infection | 1 |
|  |  |  | Vaginal infection | 1 (1) | Vaginal infection | 1 |
|  | Musculoskeletal and connective tissue | 6 (9) | Arthralgia | 2 (3) | Arthralgia | 1 |
|  |  |  |  |  | Gout | 1 |
|  |  |  | Bone pain | 1 (1) | Bone pain | 1 |
|  |  |  | Muscular pain | 2 (3) | Muscle pain | 1 |
|  |  |  |  |  | Myalgia | 1 |
|  |  |  | Musculoskeletal injury | 6 (9) | Dorsolumbar sprain and contusion | 1 |
|  |  |  |  |  | Fracture | 1 |
|  |  |  |  |  | Herniated cervical disc | 1 |
|  |  |  |  |  | Knee tendon tear and meniscus tear | 1 |
|  |  |  |  |  | Leg fracture requiring surgery | 1 |
|  |  |  |  |  | Low back pain | 1 |
|  |  |  |  |  | Tendon rupture | 1 |
|  |  |  |  |  | Traumatic fracture | 1 |
|  | Neoplasm | 66 (99) | Basal cell carcinoma | 1 (1) | Basal cell carcinoma | 1 |
|  |  |  | Lymphoma | 1 (1) | Lymphoma | 1 |
|  |  |  | Progression to ASCC | 31 (46) | ASCC Incidence | 1 |
|  |  |  |  |  | Development of anal SCC | 1 |
|  |  |  |  |  | Development of invasive anal cancer | 1 |
|  |  |  |  |  | Disease progression | 1 |
|  |  |  |  |  | Invasive cancer | 1 |
|  |  |  |  |  | Invasive perianal SCC | 1 |
|  |  |  |  |  | Invasive SCC | 1 |
|  |  |  |  |  | Progression from HGAIN to invasive anal cancer | 1 |
|  |  |  |  |  | Progression to anal cancer | 7 |
|  |  |  |  |  | Progression to anal squamous cell carcinoma | 1 |
|  |  |  |  |  | Progression to ASCC | 4 |
|  |  |  |  |  | Progression to cancer | 2 |
|  |  |  |  |  | Progression to invasion | 1 |
|  |  |  |  |  | Progression to invasive anal cancer | 1 |
|  |  |  |  |  | Progression to invasive anal carcinoma | 2 |
|  |  |  |  |  | Progression to SCC | 4 |
|  |  |  |  |  | Progression to SCCA | 1 |
|  |  |  | Disease (aHSIL) recurrence | 52 (78) | Disease free | 2 |
|  |  |  |  |  | Disease free period | 1 |
|  |  |  |  |  | Disease free rate | 1 |
|  |  |  |  |  | Ectopic recurrence | 1 |
|  |  |  |  |  | Freedom from high-grade disease | 1 |
|  |  |  |  |  | Further foci of AIN 3 | 1 |
|  |  |  |  |  | Gross recurrence | 1 |
|  |  |  |  |  | HSIL Recurrence | 2 |
|  |  |  |  |  | HSIL at last follow-up | 1 |
|  |  |  |  |  | Incident HSIL | 1 |
|  |  |  |  |  | Index lesion recurrence | 1 |
|  |  |  |  |  | Index/adjacent site recurrence | 1 |
|  |  |  |  |  | Local recurrence | 4 |
|  |  |  |  |  | Metachronous disease | 3 |
|  |  |  |  |  | Metachronous recurrence | 15 |
|  |  |  |  |  | New disease | 1 |
|  |  |  |  |  | No recurrence | 1 |
|  |  |  |  |  | Non index site recurrence | 1 |
|  |  |  |  |  | Overall recurrence | 4 |
|  |  |  |  |  | Persistent recurrence | 4 |
|  |  |  |  |  | Post-treatment HSIL recurrence | 1 |
|  |  |  |  |  | Presence of high-grade disease | 1 |
|  |  |  |  |  | Recurrence | 24 |
|  |  |  |  |  | Recurrence at the end of follow up | 1 |
|  |  |  |  |  | Recurrent HGAIN | 1 |
|  |  |  |  |  | Recurrent HSIL | 3 |
|  |  |  |  |  | Recurrent LSIL | 2 |
|  |  |  |  |  | Recurrent disease | 1 |
|  |  |  |  |  | Relapse | 1 |
|  |  |  |  |  | Time to recurrence | 10 |
|  |  |  |  |  | Treatment failure | 1 |
|  |  |  | Disease (aHSIL) response | 48 (72) | Absence of HGAIN at re-evaluation | 1 |
|  |  |  |  |  | AIN eradication | 1 |
|  |  |  |  |  | Clearance | 1 |
|  |  |  |  |  | Clearance of histological HSIL | 2 |
|  |  |  |  |  | Clinical improvement | 1 |
|  |  |  |  |  | Complete index lesion clearance (CILC) | 1 |
|  |  |  |  |  | Complete lesion resolution | 1 |
|  |  |  |  |  | Complete regression | 1 |
|  |  |  |  |  | Complete remission | 1 |
|  |  |  |  |  | Complete response | 18 |
|  |  |  |  |  | Cure | 1 |
|  |  |  |  |  | Disease progression | 2 |
|  |  |  |  |  | Downgrading | 2 |
|  |  |  |  |  | Extent response | 1 |
|  |  |  |  |  | HSIL absence | 1 |
|  |  |  |  |  | Improvement | 2 |
|  |  |  |  |  | Index lesion level complete response | 1 |
|  |  |  |  |  | Index lesion level failure | 1 |
|  |  |  |  |  | Index lesion level partial response | 1 |
|  |  |  |  |  | Initial complete response | 1 |
|  |  |  |  |  | Initial partial response | 1 |
|  |  |  |  |  | Lesion cure | 2 |
|  |  |  |  |  | No response | 7 |
|  |  |  |  |  | Overall treatment success | 1 |
|  |  |  |  |  | Overall cure | 1 |
|  |  |  |  |  | Overall response | 1 |
|  |  |  |  |  | Partial index lesion clearance (PILC) | 1 |
|  |  |  |  |  | Partial lesion resolution | 1 |
|  |  |  |  |  | Partial regression | 2 |
|  |  |  |  |  | Partial response | 17 |
|  |  |  |  |  | Pathological improvement | 1 |
|  |  |  |  |  | Patient level complete response | 1 |
|  |  |  |  |  | Patient level failure | 1 |
|  |  |  |  |  | Patient level partial response | 1 |
|  |  |  |  |  | Persistence | 14 |
|  |  |  |  |  | Persistent AIN3 | 1 |
|  |  |  |  |  | Persistent disease | 2 |
|  |  |  |  |  | Progression | 3 |
|  |  |  |  |  | Progressive disease | 2 |
|  |  |  |  |  | Recovery | 1 |
|  |  |  |  |  | Regression | 5 |
|  |  |  |  |  | Residual disease | 1 |
|  |  |  |  |  | Resolution | 1 |
|  |  |  |  |  | Response | 1 |
|  |  |  |  |  | Response to treatment | 1 |
|  |  |  |  |  | Spontaneous regression | 1 |
|  |  |  |  |  | Spontaneous resolution | 1 |
|  |  |  |  |  | Stable disease | 4 |
|  |  |  |  |  | Sustained clearance | 1 |
|  |  |  |  |  | Therapeutic failure | 1 |
|  |  |  |  |  | Time to cure | 1 |
|  |  |  |  |  | Treatment efficacy | 3 |
|  |  |  |  |  | Treatment effectiveness | 2 |
|  |  |  |  |  | Treatment failure | 4 |
|  |  |  |  |  | Treatment success | 4 |
|  |  |  |  |  | Treatment yielded cure | 1 |
|  |  |  |  |  | Worsening | 1 |
|  | Nervous system | 5 (7) | Dizziness | 3 (4) | Dizziness | 3 |
|  |  |  | Headache | 4 (6) | Headache | 4 |
|  |  |  | Paraesthesia | 1 (1) | Paraesthesia | 1 |
|  |  |  | Sleep disturbance | 2 (3) | Insomnia | 2 |
|  | Psychiatric | 7 (10) | Anxiety | 5 (7) | Anxiety | 5 |
|  |  |  |  |  | Anxiety / Depression | 1 |
|  |  |  | Depression | 1 (1) | Depression | 2 |
|  |  |  | Narcotic pain medication addiction | 1 (1) | Narcotic pain medication addiction | 1 |
|  | Renal and urinary | 4 (6) | Epididymitis | 1 (1) | Epididymitis | 1 |
|  |  |  | Kidney function | 1 (1) | Creatinine | 1 |
|  |  |  |  |  | Electrolytes | 1 |
|  |  |  |  |  | Serum urea nitrogen | 1 |
|  |  |  | Prostatitis | 1 (1) | Prostatitis | 1 |
|  |  |  | Proteinuria | 1 (1) | Proteinuria | 1 |
|  |  |  | Pyelonephritis post-pyelography | 1 (1) | Pyelonephritis post-pyelography | 1 |
|  |  |  | Renal toxicity | 1 (1) | Decompensated nephrotic syndrome with leg oedema | 1 |
|  |  |  | Urinary incontinence | 1 (1) | Urinary incontinence | 1 |
|  | Reproductive and breast system | 9 (13) | Desire for sex | 2 (3) | Desire for forms of sexual activity other than anal sexual activity | 1 |
|  |  |  |  |  | Sexual interest | 1 |
|  |  |  | Desire for sex (anal) | 1 (1) | Desire for anal sexual activity | 1 |
|  |  |  | Discomfort/pain with/following intercourse (anal) | 1 (1) | Discomfort or pain following or during passive anal sex (degree) | 1 |
|  |  |  |  |  | Discomfort or pain following or during passive anal sex (frequency) | 1 |
|  |  |  | Enjoyment of sex | 3 (4) | Enjoyment having sex | 1 |
|  |  |  |  |  | Enjoyment of forms of sexual activity other than anal sexual activity | 1 |
|  |  |  |  |  | Enjoyment of sexual activity | 1 |
|  |  |  | Enjoyment of sex (anal) | 1 (1) | Enjoyment of anal sexual activity | 1 |
|  |  |  | Erectile function | 2 (3) | Erectile dysfunction | 1 |
|  |  |  |  |  | Erection (only male) | 1 |
|  |  |  | Orgasm | 1 (1) | Orgasm | 1 |
|  |  |  | Return to sexual activity (anal) | 5 (7) | Anal receptive intercourse success | 1 |
|  |  |  |  |  | Attempt at passive anal sex | 1 |
|  |  |  |  |  | Interference with anal sex | 1 |
|  |  |  |  |  | Interruption to recommencing anal sex | 1 |
|  |  |  |  |  | Resumption of receptive anal intercourse | 1 |
|  |  |  |  |  | Successful passive anal sex | 1 |
|  |  |  | Sex satisfaction | 2 (3) | Global sexual satisfaction | 1 |
|  |  |  |  |  | Overall sex life satisfaction | 1 |
|  |  |  | Sex satisfaction (anal) | 1 (1) | Satisfactory anal sex | 1 |
|  |  |  | Sexual arousal | 1 (1) | Sexual arousal | 1 |
|  |  |  | Sexual functioning | 3 (4) | Poor sexual function | 1 |
|  |  |  |  |  | Sexual Functioning | 2 |
|  | Respiratory, thoracic and mediastinal | 5 (7) | Asthma | 1 (1) | Asthma | 1 |
|  |  |  | Bronchitis | 1 (1) | Bronchitis | 1 |
|  |  |  | Coughing | 1 (1) | Coughing | 1 |
|  |  |  | Dyspnoea | 1 (1) | Dyspnoea | 1 |
|  |  |  | Sleep apnoea | 1 (1) | Sleep apnoea | 1 |
|  |  |  | Sore throat | 1 (1) | Sore throat | 1 |
|  | Skin and subcutaneous tissue | 14 (21) | Bruising | 1 (1) | Bruising | 1 |
|  |  |  | Dry skin | 1 (1) | Dry skin | 1 |
|  |  |  | Hair loss | 1 (1) | Hair loss | 3 |
|  |  |  | Palmar Erythema | 1 (1) | Palmar erythema | 1 |
|  |  |  | Pain of skin | 1 (1) | Pain of skin | 1 |
|  |  |  | Rash | 2 (3) | Rash | 2 |
|  |  |  | Scar upper arm | 1 (1) | Scar upper arm | 1 |
|  |  |  | Small telangiectasia | 1 (1) | Small telangiectasia | 1 |
|  |  |  | Thigh Erythema | 1 (1) | Thigh warmth and erythema | 1 |
|  |  |  | Wound healing | 10 (15) | Complete mucosal healing | 1 |
|  |  |  |  |  | Failure to heal | 1 |
|  |  |  |  |  | Healed | 1 |
|  |  |  |  |  | Mucosal healing | 1 |
|  |  |  |  |  | Split skin graft success | 1 |
|  |  |  |  |  | Wound healing | 2 |
|  | Vascular | 1 (1) | Thrombophlebitis | 1 (1) | Superficial thrombophlebitis | 1 |
| Life Impact | Cognitive functioning | 2 (3) | Concentration problems | 2 (3) | Concentration problems | 1 |
|  |  |  |  |  | Difficulty Concentrating | 1 |
|  | Delivery of care | 34 (51) | Adherence/Compliance | 10 (15) | Adherence | 1 |
|  |  |  |  |  | Adherence to protocol | 1 |
|  |  |  |  |  | Adherence to treatment | 2 |
|  |  |  |  |  | Compliance | 2 |
|  |  |  |  |  | Discontinuation of treatment | 1 |
|  |  |  |  |  | Drop out | 1 |
|  |  |  |  |  | Modification of treatment regimen | 1 |
|  |  |  |  |  | Non treatment compliance | 1 |
|  |  |  |  |  | Overdosage | 1 |
|  |  |  |  |  | Reduced dose treatment completion | 1 |
|  |  |  |  |  | Treatment interruption | 2 |
|  |  |  |  |  | Treatment suspension | 1 |
|  |  |  | Duration of intervention | 1 (1) | Operating time | 1 |
|  |  |  | Procedural blood loss | 2 (3) | Procedural blood loss | 1 |
|  |  |  |  |  | Procedural bleeding | 1 |
|  |  |  | Resection margin involvement | 4 (6) | Disease-free surgical margins | 1 |
|  |  |  |  |  | Involved resection margins | 1 |
|  |  |  |  |  | Negative resection margin | 2 |
|  |  |  | Tolerability/acceptability of intervention | 28 (42) | Adverse events requiring withdrawal of treatment | 1 |
|  |  |  |  |  | Agreement to retreatment | 1 |
|  |  |  |  |  | Cessation of treatment due to side effects | 1 |
|  |  |  |  |  | Discontinuation of treatment due to side effects | 5 |
|  |  |  |  |  | Dose alteration due to adverse event | 1 |
|  |  |  |  |  | Failure to complete treatment due to side effects | 5 |
|  |  |  |  |  | Interruption to therapy due to side effects | 1 |
|  |  |  |  |  | Intolerance to imiquimod | 1 |
|  |  |  |  |  | Local tolerance | 1 |
|  |  |  |  |  | Need for dose reduction | 1 |
|  |  |  |  |  | Need for sedation | 1 |
|  |  |  |  |  | Patient satisfaction | 1 |
|  |  |  |  |  | Poor tolerance | 1 |
|  |  |  |  |  | Procedure abandonment | 1 |
|  |  |  |  |  | Safety | 1 |
|  |  |  |  |  | Safety of surgical treatment | 1 |
|  |  |  |  |  | Safety of treatment | 2 |
|  |  |  |  |  | Tolerability | 4 |
|  |  |  |  |  | Tolerance | 1 |
|  |  |  |  |  | Treatment discontinuation for adverse effects | 2 |
|  |  |  |  |  | Treatment interruption due to side effects | 3 |
|  |  |  |  |  | Treatment modification for side effects | 1 |
|  |  |  |  |  | Treatment reduction due to side effects | 1 |
|  |  |  |  |  | Treatment reduction for moderate or severe perianal irritation or pain | 1 |
|  |  |  |  |  | Treatment reduction for side effects | 1 |
|  |  |  |  |  | Treatment switch due to side effects | 1 |
|  | Emotional functioning/wellbeing | 4 (6) | Anxious symptoms | 2 (3) | Anxious symptoms | 1 |
|  |  |  |  |  | Calmness | 1 |
|  |  |  |  |  | Nervousness | 1 |
|  |  |  | Depressive symptoms | 2 (3) | Depressive symptoms | 1 |
|  |  |  |  |  | Downheartedness | 1 |
|  |  |  |  |  | Happiness | 1 |
|  |  |  | Emotional wellbeing | 1 (1) | Emotional wellbeing | 1 |
|  |  |  | Mood change | 2 (3) | Irritability | 1 |
|  |  |  |  |  | Mood swings | 2 |
|  |  |  | Psychological symptoms | 1 (1) | Psychological symptoms | 1 |
|  |  |  | Worried about condition getting worse | 1 (1) | Worried about condition getting worse | 1 |
|  | Global quality of life | 2 (3) | Quality of life | 2 (3) | Quality of life | 2 |
|  | Perceived health status | 3 (4) | Symptom response | 3 (4) | Asymptomatic | 1 |
|  |  |  |  |  | Physical symptoms | 1 |
|  |  |  |  |  | Resolution of HSIL symptoms | 1 |
|  | Physical functioning | 5 (7) | Daily activities | 5 (7) | Interference with bathing or dressing yourself | 1 |
|  |  |  |  |  | Interference with lifting or carrying groceries | 1 |
|  |  |  |  |  | Interference with moderate activities (e.g., moving a table, pushing a vacuum cleaner) | 1 |
|  |  |  |  |  | Interference with vigorous activities (e.g., running, lifting heavy objects) | 1 |
|  |  |  |  |  | Loss of ADLs | 1 |
|  |  |  |  |  | Problems participating in leisure activities (e.g., watching television, relaxing) | 1 |
|  |  |  |  |  | Problems taking care of myself (e.g., bathing, dressing, shaving) | 1 |
|  |  |  |  |  | Problems with completing daily household chores (e.g., cleaning, cooking, laundry, house maintenance) | 1 |
|  |  |  |  |  | Self-care | 1 |
|  |  |  |  |  | Time to resumption of normal activities | 1 |
|  |  |  |  |  | Time to return to normal activities | 1 |
|  |  |  |  |  | Usual activities | 1 |
|  |  |  | Mobility | 2 (3) | Interference with bending, kneeling, or stooping | 1 |
|  |  |  |  |  | Interference with climbing one flight of stairs | 1 |
|  |  |  |  |  | Interference with climbing several flights of stairs | 1 |
|  |  |  |  |  | Interference with walking more than a mile | 1 |
|  |  |  |  |  | Interference with walking one block | 1 |
|  |  |  |  |  | Interference with walking several blocks | 1 |
|  |  |  |  |  | Mobility | 1 |
|  |  |  |  |  | Problems with my physical ability to move around | 1 |
|  |  |  | Physical functioning | 2 (3) | Physical functioning | 2 |
|  |  |  | Sitting | 1 (1) | Problems with sitting | 1 |
|  | Role functioning | 2 (3) | Ability to work | 2 (3) | Inability to work | 1 |
|  |  |  |  |  | Problems with work productivity | 1 |
|  | Social functioning | 2 (3) | Intimate relationships | 1 (1) | Problems with intimate relationships | 1 |
|  |  |  | Social activities | 2 (3) | Interference with social activities (extent) | 1 |
|  |  |  |  |  | Interference with social activities (frequency) | 1 |
|  |  |  |  |  | Problems participating in social activities (e.g., going out to eat, visiting friends) | 1 |
|  |  |  | Social functioning | 1 (1) | Social functioning | 1 |
| Resource use | Hospital | 1 (1) | Representation/rehospitalisation | 1 (1) | Care sought for bleeding | 1 |
|  | Need for further intervention | 28 (42) | Analgesic use | 8 (12) | Analgesia control at discharge | 1 |
|  |  |  |  |  | Analgesic requirement | 1 |
|  |  |  |  |  | Analgesic use | 2 |
|  |  |  |  |  | Narcotic use | 1 |
|  |  |  |  |  | Pain control | 3 |
|  |  |  | Closure of stoma | 1 (1) | Temporary stoma closure | 1 |
|  |  |  | Flap reconstruction | 1 (1) | Flap reconstruction | 1 |
|  |  |  | Intervention for adverse effects | 1 (1) | Treatment for side effects | 1 |
|  |  |  | Intervention for bleeding | 5 (7) | Intervention for bleeding | 3 |
|  |  |  |  |  | Need for endoscopy/surgery for bleeding | 1 |
|  |  |  |  |  | Need for transfusion | 1 |
|  |  |  |  |  | Reoperation for bleeding | 1 |
|  |  |  | Number of treatments to response | 4 (6) | Number of treatments to clearance | 1 |
|  |  |  |  |  | Number of treatments to response | 1 |
|  |  |  |  |  | Number of procedures until radicality | 1 |
|  |  |  |  |  | Treatment responsiveness | 1 |
|  |  |  | Reoperation for abscess | 1 (1) | Reoperation for abscess | 1 |
|  |  |  | Retreatment | 16 (24) | Additional IRC treatment | 1 |
|  |  |  |  |  | Alternative treatment | 2 |
|  |  |  |  |  | Further ablation | 1 |
|  |  |  |  |  | Further procedure | 1 |
|  |  |  |  |  | Further treatment | 3 |
|  |  |  |  |  | Further treatment for persistence/recurrence | 1 |
|  |  |  |  |  | Need for alternative ablation laser therapy | 1 |
|  |  |  |  |  | Need for further ablation | 1 |
|  |  |  |  |  | Need for further excision | 1 |
|  |  |  |  |  | Need for further treatment | 1 |
|  |  |  |  |  | Number of treatment sessions | 1 |
|  |  |  |  |  | Repeat ablation | 1 |
|  |  |  |  |  | Retreatment | 2 |
|  |  |  |  |  | Retreatment after initial treatment | 1 |
|  |  |  |  |  | Retreatment after repeat treatment | 1 |
|  |  |  | Stoma formation | 5 (7) | Colostomy | 1 |
|  |  |  |  |  | Defunctioning colostomy | 1 |
|  |  |  |  |  | Permanent colostomy | 1 |
|  |  |  |  |  | Stoma formation | 2 |
| Death | Mortality/Survival | 10 (15) | Death | 2 (3) | Death | 2 |
|  |  |  | Death from metastatic anal cancer | 1 (1) | Death from metastatic anal cancer | 1 |
|  |  |  | Death unrelated to treatment effect | 1 (1) | Death unrelated to treatment effect | 1 |
|  |  |  | Disease free survival | 5 (7) | HSIL-free Survival | 1 |
|  |  |  |  |  | Lesion free survival | 2 |
|  |  |  |  |  | Recurrence-free survival | 2 |
|  |  |  | Overall survival | 1 (1) | Crude survival rate | 1 |
|  |  |  | Treatment related death | 2 (3) | Treatment related death | 2 |
| Adverse events | Adverse events | 37 (55) | Adverse events | 38 (57) | Adverse effects | 1 |
|  |  |  |  |  | Adverse event symptom length | 1 |
|  |  |  |  |  | Adverse events | 11 |
|  |  |  |  |  | Complication | 1 |
|  |  |  |  |  | Duration of adverse effects | 3 |
|  |  |  |  |  | Duration of adverse events | 1 |
|  |  |  |  |  | Incidence of adverse events | 1 |
|  |  |  |  |  | Life threatening complications | 1 |
|  |  |  |  |  | Local adverse effects | 1 |
|  |  |  |  |  | Major complication | 3 |
|  |  |  |  |  | Mild side effects | 1 |
|  |  |  |  |  | Minor adverse events | 1 |
|  |  |  |  |  | Minor complication | 2 |
|  |  |  |  |  | Serious adverse effects | 1 |
|  |  |  |  |  | Serious adverse events | 13 |
|  |  |  |  |  | Serious local adverse effects | 1 |
|  |  |  |  |  | Severity of adverse events | 1 |
|  |  |  |  |  | Side effect duration | 2 |
|  |  |  |  |  | Side effects | 4 |
|  |  |  |  |  | Significant complications | 2 |
|  |  |  |  |  | Significant late complications | 1 |
|  |  |  |  |  | Systemic adverse effects | 1 |
|  |  |  |  |  | Time to local adverse event | 1 |
|  |  |  |  |  | Time to resolution of local adverse event | 1 |

Table S4. Outcomes extracted from quality of life/functioning patient-reported outcome measurement (PROM) tools.

| **Core area** | **Outcome domain** | **Standardised outcome term** | **Measurement instrument (number of studies using instrument)** | | | | | |
| --- | --- | --- | --- | --- | --- | --- | --- | --- |
|  |  |  | **A-HRSI (1)** | **EQ5D (1)** | **Female sexual function index (1)** | **Index of Erectile function (1)** | **MGH-SFQ (1)** | **SF-36 (1)** |
| Physiological/clinical | Gastrointestinal | Anal bleeding | 1 |  |  |  |  |  |
|  |  | Anal discharge | 1 |  |  |  |  |  |
|  |  | Anal itch | 1 |  |  |  |  |  |
|  |  | Anal pain | 3 |  |  |  |  |  |
|  |  | Constipation | 1 |  |  |  |  |  |
|  |  | Faecal urgency | 1 |  |  |  |  |  |
|  | General | Unspecified (bodily) pain | 1 | 1 |  |  |  | 3* |
|  |  | General health |  |  |  |  |  | 6* |
|  | Psychiatric | Anxiety / Depression |  | 1 |  |  |  |  |
|  | Reproductive and breast system | Desire for sex | 1 |  |  |  | 1 |  |
|  |  | Desire for sex (anal) | 1 |  |  |  |  |  |
|  |  | Discomfort/pain with/following intercourse (anal) |  |  | 2 |  |  |  |
|  |  | Enjoyment of sex | 1 |  |  | 1 |  |  |
|  |  | Enjoyment of sex (anal) | 1 |  |  |  |  |  |
|  |  | Erectile function |  |  |  |  | 1 |  |
|  |  | Orgasm |  |  |  |  | 1 |  |
|  |  | Return to sexual activity (anal) |  |  |  | 2 |  |  |
|  |  | Sex satisfaction |  |  |  | 1 | 1 |  |
|  |  | Sex satisfaction (anal) |  |  |  | 1 |  |  |
|  |  | Sexual arousal |  |  |  |  | 1 |  |
|  |  | Sexual functioning |  |  |  |  | 1* |  |
|  |  |  |  |  |  |  |  |  |
| Life impact | Cognitive functioning | Concentration problems | 1 |  |  |  |  |  |
|  | Emotional functioning/wellbeing | Anxious symptoms | 1 |  |  |  |  |  |
|  |  | Depressive symptoms | 1 |  |  |  |  |  |
|  |  | Emotional wellbeing |  |  |  |  |  | 1* |
|  |  | Psychological symptoms | 1* |  |  |  |  |  |
|  |  | Worried about condition getting worse | 1 |  |  |  |  |  |
|  | Perceived health status | Symptom response | 1* |  |  |  |  |  |
|  | Physical functioning | Daily activities | 3 | 2 |  |  |  | 4 |
|  |  | Mobility | 1 | 1 |  |  |  | 6 |
|  |  | Physical functioning | 1* |  |  |  |  | 1* |
|  |  | Sitting | 1 |  |  |  |  |  |
|  | Role functioning | Ability to work | 1 |  |  |  |  |  |
|  | Social functioning | Intimate relationships | 1 |  |  |  |  |  |
|  |  | Social activities | 1 |  |  |  |  | 2 |
|  |  | Social functioning |  |  |  |  |  | 1* |

Numbers in cells show the number of verbatim question items summarised into each standardised outcome term

Outcomes were extracted at both the domain level, representing composite scores from multiple questions within a specific construct, and at the individual item level, for single questions that were of direct clinical or research interest. Those numbers marked with * include domain level outcomes.

Abbreviations: A-HRSI, ANCHOR Health-Related Symptom Index; EQ5D, EuroQol 5 Dimensions; MGH-SFQ, Massachusetts General Hospital-Sexual Functioning Questionnaire; SF-36, 36-Item Short Form Survey.

References

1. Scholefield JH, Ogunbiyi OA, Smith JH, et al. Treatment of anal intraepithelial neoplasia. Br J Surg. 1994;81(8):1238-40. 10.1002/bjs.1800810855

2. Marchesa P, Fazio VW, Oliart S, et al. Perianal Bowen's disease: a clinicopathologic study of 47 patients. Dis Colon Rectum. 1997;40(11):1286-93. 10.1007/BF02050810

3. Brown SR, Skinner P, Tidy J, et al. Outcome after surgical resection for high-grade anal intraepithelial neoplasia (Bowen's disease). Br J Surg. 1999;86(8):1063-6. 10.1046/j.1365-2168.1999.01184.x

4. Klencke B, Matijevic M, Urban RG, et al. Encapsulated plasmid DNA treatment for human papillomavirus 16-associated anal dysplasia: a Phase I study of ZYC101. Clin Cancer Res. 2002;8(5):1028-37.

5. Chang GJ, Berry JM, Jay N, et al. Surgical treatment of high-grade anal squamous intraepithelial lesions: a prospective study. Dis Colon Rectum. 2002;45(4):453-8. 10.1007/s10350-004-6219-8

6. Webber J, Fromm D. Photodynamic therapy for carcinoma in situ of the anus. Arch Surg. 2004;139(3):259-61. 10.1001/archsurg.139.3.259

7. Goldstone SE, Kawalek AZ, Huyett JW. Infrared coagulatorTM: A useful tool for treating anal squamous intraepithelial lesions. Diseases of the Colon and Rectum. 2005;48(5):1042-54. <https://dx.doi.org/10.1007/s10350-004-0889-0>

8. Scholefield JH, Castle MT, Watson NF. Malignant transformation of high-grade anal intraepithelial neoplasia. Br J Surg. 2005;92(9):1133-6. 10.1002/bjs.4994

9. Graham BD, Jetmore AB, Foote JE, et al. Topical 5-fluorouracil in the management of extensive anal Bowen's disease: a preferred approach. Dis Colon Rectum. 2005;48(3):444-50. 10.1007/s10350-004-0901-8

10. Palefsky JM, Michael Berry J, Jay N, et al. A trial of SGN-00101 (HspE7) to treat high-grade anal intraepithelial neoplasia in HIV-positive individuals. AIDS. 2006;20(8):1151-5. <https://dx.doi.org/10.1097/01.aids.0000226955.02719.26>

11. Wieland U, Brockmeyer NH, Weissenborn SJ, et al. Imiquimod treatment of anal intraepithelial neoplasia in HIV-positive men. Arch Dermatol. 2006;142(11):1438-44. 10.1001/archderm.142.11.1438

12. Watson AJM, Smith BB, Whitehead MR, et al. Malignant progression of anal intra-epithelial neoplasia. ANZ Journal of Surgery. 2006;76(8):715-7. <https://dx.doi.org/10.1111/j.1445-2197.2006.03837.x>

13. Goldstone SE, Hundert JS, Huyett JW. Infrared coagulator ablation of high-grade anal squamous intraepithelial lesions in HIV-negative males who have sex with males. Diseases of the Colon and Rectum. 2007;50(5):565-75. <https://dx.doi.org/10.1007/s10350-006-0874-x>

14. Pineda CE, Berry JM, Jay N, et al. High resolution anoscopy in the planned staged treatment of anal squamous intraepithelial lesions in HIV-negative patients. J Gastrointest Surg. 2007;11(11):1410-5; discussion 5-6. 10.1007/s11605-007-0262-4

15. Nathan M, Hickey N, Mayuranathan L, et al. Treatment of anal human papillomavirus-associated disease: a long term outcome study. Int J STD AIDS. 2008;19(7):445-9. 10.1258/ijsa.2007.007290

16. Cranston RD, Hirschowitz SL, Cortina G, et al. A retrospective clinical study of the treatment of high-grade anal dysplasia by infrared coagulation in a population of HIV-positive men who have sex with men. Int J STD AIDS. 2008;19(2):118-20. 10.1258/ijsa.2007.005665

17. Kreuter A, Potthoff A, Brockmeyer NH, et al. Imiquimod leads to a decrease of human papillomavirus DNA and to a sustained clearance of anal intraepithelial neoplasia in HIV-infected men. J Invest Dermatol. 2008;128(8):2078-83. 10.1038/jid.2008.24

18. Pineda CE, Berry JM, Jay N, et al. High-resolution anoscopy targeted surgical destruction of anal high-grade squamous intraepithelial lesions: a ten-year experience. Dis Colon Rectum. 2008;51(6):829-35; discussion 35-7. 10.1007/s10350-008-9233-4

19. Stier EA, Goldstone SE, Berry JM, et al. Infrared coagulator treatment of high-grade anal dysplasia in HIV-infected individuals: an AIDS malignancy consortium pilot study. J Acquir Immune Defic Syndr. 2008;47(1):56-61. 10.1097/QAI.0b013e3181582d93

20. Singh JC, Kuohung V, Palefsky JM. Efficacy of trichloroacetic acid in the treatment of anal intraepithelial neoplasia in HIV-positive and HIV-negative men who have sex with men. J Acquir Immune Defic Syndr. 2009;52(4):474-9. 10.1097/QAI.0b013e3181bc0f10

21. Fox PA, Nathan M, Francis N, et al. A double-blind, randomized controlled trial of the use of imiquimod cream for the treatment of anal canal high-grade anal intraepithelial neoplasia in HIV-positive MSM on HAART, with long-term follow-up data including the use of open-label imiquimod. AIDS. 2010;24(15):2331-5. 10.1097/QAD.0b013e32833d466c

22. Richel O, Wieland U, De Vries HJC, et al. Topical 5-fluorouracil treatment of anal intraepithelial neoplasia in human immunodeficiency virus-positive men. British Journal of Dermatology. 2010;163(6):1301-7. <https://dx.doi.org/10.1111/j.1365-2133.2010.09982.x>

23. Snyder SM, Siekas L, Aboulafia DM. Initial Experience with Topical Fluorouracil for Treatment of HIV-Associated Anal Intraepithelial Neoplasia. J Int Assoc Physicians AIDS Care (Chic). 2011;10(2):83-8. 10.1177/1545109710382578

24. Weis SE, Vecino I, Pogoda JM, et al. Treatment of high-grade anal intraepithelial neoplasia with infrared coagulation in a primary care population of HIV-infected men and women. Dis Colon Rectum. 2012;55(12):1236-43. 10.1097/DCR.0b013e31826d5cb5

25. Marks DK, Goldstone SE. Electrocautery ablation of high-grade anal squamous intraepithelial lesions in HIV-negative and HIV-positive men who have sex with men. J Acquir Immune Defic Syndr. 2012;59(3):259-65. 10.1097/QAI.0b013e3182437469

26. Van Der Snoek EM, Den Hollander JC, Aans JB, et al. Photodynamic therapy with systemic meta-tetrahydroxyphenylchlorin in the treatment of anal intraepithelial neoplasia, grade 3. Lasers in Surgery and Medicine. 2012;44(8):637-44. <https://dx.doi.org/10.1002/lsm.22062>

27. Macaya A, Munoz-Santos C, Balaguer A, et al. Interventions for anal canal intraepithelial neoplasia. Cochrane Database Syst Rev. 2012;12(12):CD009244. 10.1002/14651858.CD009244.pub2

28. Assoumou SA, Panther LA, Mayer KH. Treatment of high-grade anal dysplasia in high-risk patients: Outcome at an urban community health centre. International Journal of STD and AIDS. 2013;24(2):134-8. <https://dx.doi.org/10.1177/0956462412472298>

29. Stier EA, Goldstone SE, Einstein MH, et al. Safety and efficacy of topical cidofovir to treat high-grade perianal and vulvar intraepithelial neoplasia in HIV-positive men and women. AIDS. 2013;27(4):545-51. 10.1097/QAD.0b013e32835a9b16

30. Sirera G, Videla S, Pinol M, et al. Long-term effectiveness of infrared coagulation for the treatment of anal intraepithelial neoplasia grades 2 and 3 in HIV-infected men and women. AIDS. 2013;27(6):951-9. 10.1097/QAD.0b013e32835e06c1

31. Richel O, de Vries HJC, van Noesel CJM, et al. Comparison of imiquimod, topical fluorouracil, and electrocautery for the treatment of anal intraepithelial neoplasia in HIV-positive men who have sex with men: An open-label, randomised controlled trial. The Lancet Oncology. 2013;14(4):346-53. <https://dx.doi.org/10.1016/S1470-2045%2813%2970067-6>

32. Cranston RD, Baker JR, Liu Y, et al. Topical application of trichloroacetic acid is efficacious for the treatment of internal anal high-grade squamous intraepithelial lesions in HIV-positive men. Sex Transm Dis. 2014;41(7):420-6. 10.1097/OLQ.0000000000000145

33. Smulian A, Moore D, Robertson J, et al. Phase i study demonstrates safety and tolerability of radiofrequency ablation (RFA) of the anal mucosa. HIV Clinical Trials. 2014;15(1):36-44. <https://dx.doi.org/10.1310/hct1501-36>

34. Goldstone SE, Johnstone AA, Moshier EL. Long-term outcome of ablation of anal high-grade squamous intraepithelial lesions: recurrence and incidence of cancer. Dis Colon Rectum. 2014;57(3):316-23. 10.1097/DCR.0000000000000058

35. van der Snoek EM, den Hollander JC, van der Ende ME. Imiquimod 5% cream for five consecutive days a week in an HIV-infected observational cohort up to 32 weeks in the treatment of high-grade squamous intraepithelial lesions. Sex Transm Infect. 2015;91(4):245-7. 10.1136/sextrans-2014-051810

36. Johnstone AA, Silvera R, Goldstone SE. Targeted ablation of perianal high-grade dysplasia in men who have sex with men: an alternative to mapping and wide local excision. Dis Colon Rectum. 2015;58(1):45-52. 10.1097/DCR.0000000000000241

37. Sendagorta E, Bernardino JI, Álvarez-Gallego M, et al. Topical cidofovir to treat high-grade anal intraepithelial neoplasia in HIV-infected patients: a pilot clinical trial. Aids. 2016;30(1):75-82. 10.1097/qad.0000000000000886

38. Siegenbeek van Heukelom ML, Richel O, Nieuwkerk PT, et al. Health-Related Quality of Life and Sexual Functioning of HIV-Positive Men Who Have Sex With Men Who Are Treated for Anal Intraepithelial Neoplasia. Dis Colon Rectum. 2016;59(1):42-7. 10.1097/dcr.0000000000000511

39. Burgos J, Curran A, Landolfi S, et al. The effectiveness of electrocautery ablation for the treatment of high-grade anal intraepithelial neoplasia in HIV-infected men who have sex with men. HIV Med. 2016;17(7):524-31. 10.1111/hiv.12352

40. Willeford WG, Barroso L, Keller J, et al. Anal Dysplasia Screening and Treatment in a Southern Human Immunodeficiency Virus Clinic. Sex Transm Dis. 2016;43(8):479-82. 10.1097/OLQ.0000000000000475

41. Willems N, Libois A, Nkuize M, et al. Treatment of anal dysplasia in HIV-positive men who have sex with men in a large AIDS reference centre. Acta Clin Belg. 2017;72(1):29-35. 10.1080/17843286.2015.1116725

42. Goldstone RN, Hasan SR, Drury S, et al. A trial of radiofrequency ablation for anal intraepithelial neoplasia. Int J Colorectal Dis. 2017;32(3):357-65. 10.1007/s00384-016-2679-2

43. Goldstone RN, Hasan SR, Goldstone SE. Brief Report: Radiofrequency Ablation Therapy for Anal Intraepithelial Neoplasia: Results From a Single-Center Prospective Pilot Study in HIV+ Participants. J Acquir Immune Defic Syndr. 2017;76(4):e93-e7. 10.1097/QAI.0000000000001535

44. Cranston RD, Baker JR, Siegel A, et al. A pilot study of the immunologic, virologic, and pathologic consequences of intra-anal 5% imiquimod in HIV-1-infected men with high-grade squamous intraepithelial lesions. Diseases of the Colon and Rectum. 2018;61(3):298-305. <https://dx.doi.org/10.1097/DCR.0000000000000991>

45. Siegenbeek van Heukelom ML, Gosens KCM, Prins JM, et al. Cryotherapy for Intra- and Perianal High-Grade Squamous Intraepithelial Lesions in HIV-Positive Men who have Sex with Men. Am J Clin Dermatol. 2018;19(1):127-32. 10.1007/s40257-017-0311-z

46. de Pokomandy A, Rouleau D, Lalonde R, et al. Argon plasma coagulation treatment of anal high-grade squamous intraepithelial lesions in men who have sex with men living with HIV: results of a 2-year prospective pilot study. HIV Medicine. 2018;19(2):81-9. <https://dx.doi.org/10.1111/hiv.12544>

47. Burgos J, Martin-Castillo M, Landolfi S, et al. Effectiveness of trichloroacetic acid vs. Electrocautery ablation for the treatment of anal high-grade squamous intraepithelial lesion in HIV-infected patients. Journal of Acquired Immune Deficiency Syndromes. 2018;79(5):612-6. <https://dx.doi.org/10.1097/QAI.0000000000001847>

48. Goldstone SE, Lensing SY, Stier EA, et al. A Randomized Clinical Trial of Infrared Coagulation Ablation Versus Active Monitoring of Intra-anal High-grade Dysplasia in Adults With Human Immunodeficiency Virus Infection: An AIDS Malignancy Consortium Trial. Clin Infect Dis. 2019;68(7):1204-12. 10.1093/cid/ciy615

49. Corral J, Pares D, Garcia-Cuyas F, et al. Recurrence risk factors after infrarred coagulation for high-grade anal intraepithelial neoplasia. Diseases of the Colon and Rectum. 2019;62(6):e264-e5. <https://dx.doi.org/10.1097/DCR.0000000000001415>

50. Gaisa MM, Liu Y, Deshmukh AA, et al. Electrocautery ablation of anal high-grade squamous intraepithelial lesions: Effectiveness and key factors associated with outcomes. Cancer. 2020;126(7):1470-9. 10.1002/cncr.32581

51. Stier EA, Abbasi W, Agyemang AF, et al. Brief Report: Recurrence of Anal High-Grade Squamous Intraepithelial Lesions Among Women Living With HIV. J Acquir Immune Defic Syndr. 2020;84(1):66-9. 10.1097/qai.0000000000002304

52. Brogden DRL, Walsh U, Pellino G, et al. Evaluating the efficacy of treatment options for anal intraepithelial neoplasia: a systematic review. Int J Colorectal Dis. 2021;36(2):213-26. 10.1007/s00384-020-03740-6

53. Corral J, Parés D, García-Cuyás F, et al. Incidence of Recurrent High-Grade Anal Dysplasia in HIV-1-Infected Men and Women Following Infrared Coagulation Ablation: A Retrospective Cohort Study. Pathogens. 2021;10(2). 10.3390/pathogens10020208

54. Vergara-Fernandez O, Solórzano-Vicuña D, Coss-Adame E, et al. Outcomes of radiofrequency ablation for anal high-grade squamous intraepithelial lesions. Tech Coloproctol. 2021;25(6):701-7. 10.1007/s10151-020-02379-3

55. Fuertes I, Cranston R, de Lazzari E, et al. Response factors associated with electrocautery treatment of intra-anal high-grade squamous intraepithelial lesions in a population of HIV-positive men who have sex with men. Int J STD AIDS. 2021;32(11):1052-9. 10.1177/09564624211017005

56. Hidalgo-Tenorio C, Garcia-Martinez CM, Pasquau J, et al. Risk factors for >=high-grade anal intraepithelial lesions in MSM living with HIV and the response to topical and surgical treatments. PLoS ONE. 2021;16(2 February):e0245870. <https://dx.doi.org/10.1371/journal.pone.0245870>

57. Palefsky JM, Lee JY, Jay N, et al. Treatment of Anal High-Grade Squamous Intraepithelial Lesions to Prevent Anal Cancer. New England Journal of Medicine. 2022;386(24):2273-82. <https://dx.doi.org/10.1056/NEJMoa2201048>

58. Fuertes I, Chivite I, Cranston RD, et al. Short-term effectiveness and tolerability of carbon dioxide laser for anal high-grade squamous intraepithelial lesions in individuals living with HIV. Int J STD AIDS. 2022;33(7):709-17. 10.1177/09564624221100069

59. Martinez CG, Pilar Hernandez Casanovas M, Vilaplana LS, et al. Our experience in the short-term diagnostic-therapeutic management of the patient with anal dysplasia. Cirugia espanola. 2022. <https://dx.doi.org/10.1016/j.cireng.2022.09.025>

60. Burgos J, Campany D, Garcia J, et al. Effectiveness of topical cidofovir for treatment of refractory anal high-grade squamous intraepithelial lesion. AIDS. 2023;37(9):1425-9. 10.1097/QAD.0000000000003591

61. Gosens KCM, van der Burg SH, Welters MJP, et al. Therapeutic Vaccination against Human Papillomavirus Type 16 for the Treatment of High-Grade Anal Intraepithelial Neoplasia in HIV+ Men. Clin Cancer Res. 2023;29(20):4109-17. 10.1158/1078-0432.CCR-22-3361

62. Fang SH, Plesa M, Carchman EH, et al. A phase I study of intra-anal artesunate (suppositories) to treat anal high-grade squamous intraepithelial lesions. PloS one. 2023;18(12):e0295647. 10.1371/journal.pone.0295647

63. Singhartinger F, Gantschnigg A, Holzinger J, et al. Safety, feasibility, and short-term-outcome of anal endoscopic submucosal dissection for anal intraepithelial neoplasia: an option for focal lesions? Techniques in Coloproctology. 2024;28(1):18. <https://dx.doi.org/10.1007/s10151-023-02896-x>

64. Borch-Johnsen P, Nygren J, Schmidt PT. Endoscopic underwater detection and resection of anal squamous intraepithelial lesions in non-anesthetized patients - a feasibility study and comparison with standard surgical treatment. Scandinavian journal of gastroenterology. 2024;59(2):232-8. 10.1080/00365521.2023.2268229

65. Burgos J, Curran A, Garcia J, et al. Effectiveness of trichloroacetic acid versus electrocautery for the treatment of anal high-grade squamous intraepithelial lesions in persons with HIV. Infect Dis (Lond). 2024;56(4):299-307. 10.1080/23744235.2024.2303021

66. Kitamura H, Ando N, Mizushima D, et al. Electrocautery ablation therapy for anal intraepithelial carcinoma with high-resolution anoscopy. Journal of infection and chemotherapy : official journal of the Japan Society of Chemotherapy. 2025;31(3):102608. 10.1016/j.jiac.2025.102608

67. Burgos J, Curran A, Garcia J, et al. Effectiveness of electrocautery, topical cidofovir and topical sinecatechins for the Treatment of Anal High-grade Squamous Intraepithelial Lesions in Persons with HIV: an open-label, randomized controlled trial. Clinical infectious diseases : an official publication of the Infectious Diseases Society of America. 2025:ciaf086. 10.1093/cid/ciaf086

68. Atkinson TM, Mazumdar M, Van Hyfte G, et al. Health-Related Quality of Life for Persons Treated or Monitored for Anal High-Grade Squamous Intraepithelial Lesions (AMC-A01). JCO Oncol Pract. 2025:OP2400830. 10.1200/OP-24-00830

69. Gallio N, Preti M, Casetta E, et al. Imiquimod for Anal High Grade Intraepithelial Neoplasia: A Systematic Review. Curr Oncol Rep. 2025. 10.1007/s11912-025-01675-1
