# Supplementary material for: Outcomes reported in studies of anal high-grade squamous intraepithelial lesions treatments: systematic review
Source: BJS Open. 2026 Jul 4;10(4):zrag061. doi: 10.1093/bjsopen/zrag061 (PMC13332405; doi:10.1093/bjsopen/zrag061)
Supplement: zrag061_Supplementary_Data [file zrag061_supplementary_data.zip › _COrSIcA_ _ PRISMA-2009-checklist.doc]

| **Section/topic** | **#** | **Checklist item** | **Reported on page #** |
| --- | --- | --- | --- |
| **TITLE** | | |  |
| Title | 1 | Identify the report as a systematic review, meta-analysis, or both. | **Page:** Title page (p.1)  **Text:** *“Systematic Review of Outcomes Reported in Treatment Studies of Anal High-Grade Squamous Intraepithelial Lesions to Inform Core Outcome Set Development.”* |
| **ABSTRACT** | | |  |
| Structured summary | 2 | Provide a structured summary including, as applicable: background; objectives; data sources; study eligibility criteria, participants, and interventions; study appraisal and synthesis methods; results; limitations; conclusions and implications of key findings; systematic review registration number. | **Page:** Abstract (p.4)  **Text:** *“This systematic review summarises outcomes reported in studies evaluating treatments for anal high-grade squamous intraepithelial lesions. Databases searched included MEDLINE, Embase, CINAHL, and the Cochrane Library (inception to June 2025). Eligible studies evaluated any anal HSIL treatment in any population. Verbatim outcomes were extracted and mapped to standardised outcome terms using the COMET taxonomy.”* |
| **INTRODUCTION** | | |  |
| Rationale | 3 | Describe the rationale for the review in the context of what is already known. | **Page:** Introduction (p.5)  **Text:** *“Interpretation and comparison of existing studies are hampered by heterogeneous outcome definitions and inconsistent reporting, which limit evidence synthesis and weaken trial conclusions. High-quality interventional studies that measure clinically meaningful outcomes are needed. A core outcome set (COS) is a key enabler. A COS provides a minimum set of outcomes that should be measured and reported in all trials for a given condition. Developed with input from key stakeholders, implementation of a COS will standardise outcome reporting, enable robust comparison across trials, and ensure that outcomes are clinically meaningful to both patients and clinicians.”* |
| Objectives | 4 | Provide an explicit statement of questions being addressed with reference to participants, interventions, comparisons, outcomes, and study design (PICOS). | **Page:** End of Introduction (p.5)  **Text:**  *“As the first stage in COS development, we undertook a systematic review to summarise the outcomes reported in studies evaluating treatments for anal HSIL.”* |
| **METHODS** | | |  |
| Protocol and registration | 5 | Indicate if a review protocol exists, if and where it can be accessed (e.g., Web address), and, if available, provide registration information including registration number. | **Page:** Methods (pp. 5-6)  **Text:**  “This systematic review was registered prospectively with the International Prospective Register of Systematic Reviews (PROSPERO CRD 42023429661) and is reported in accordance with the Preferred Reporting Items for Systematic Reviews and Meta-Analyses (PRISMA) guidelines.” |
| Eligibility criteria | 6 | Specify study characteristics (e.g., PICOS, length of follow-up) and report characteristics (e.g., years considered, language, publication status) used as criteria for eligibility, giving rationale. | **Page:** Methods – Eligibility criteria (p.6)  **Text:** *“Peer-reviewed systematic reviews, RCTs, prospective and retrospective cohort studies in adult populations (≥18 years), evaluating any treatment intervention for anal HSIL…with full text available in English were eligible for inclusion.”* |
| Information sources | 7 | Describe all information sources (e.g., databases with dates of coverage, contact with study authors to identify additional studies) in the search and date last searched. | **Page:** Methods – Search strategy (p.6)  **Text:** *“MEDLINE, Embase, The Cochrane Library, and CINAHL databases were searched from inception until June 2025.”* |
| Search | 8 | Present full electronic search strategy for at least one database, including any limits used, such that it could be repeated. | **Page:** Methods – Search strategy (p.6); Supplementary Table S1  **Text:**  *“The full search strategy was developed with the support of a University of Manchester librarian. An example MEDLINE search strategy is provided in Table S1.”* |
| Study selection | 9 | State the process for selecting studies (i.e., screening, eligibility, included in systematic review, and, if applicable, included in the meta-analysis). | **Page:** Methods – Assessment of eligibility (p.6)  **Text:** *“Titles and abstracts were screened independently by two review authors…Discrepancies were resolved through discussion. Full-text screening was undertaken for all potentially eligible studies.”* |
| Data collection process | 10 | Describe method of data extraction from reports (e.g., piloted forms, independently, in duplicate) and any processes for obtaining and confirming data from investigators. | **Page:** Methods – Data extraction (p.6–7)  **Text:** *“Data were extracted into a Microsoft Excel spreadsheet. For quality assurance, two authors extracted data from the same five articles until concordance was achieved.”* |
| Data items | 11 | List and define all variables for which data were sought (e.g., PICOS, funding sources) and any assumptions and simplifications made. | **Page:** Methods – Data extraction (p.7)  **Text:** *“All outcomes reported by a study were extracted verbatim along with accompanying definitions, measurement tools, and timepoints.”*  *“Baseline characteristics recorded included study design, intervention, participant characteristics, disease extent, and length of follow-up.”* |
| Risk of bias in individual studies | 12 | Describe methods used for assessing risk of bias of individual studies (including specification of whether this was done at the study or outcome level), and how this information is to be used in any data synthesis. | **Page:** Methods – Assessment of bias (p.7)  **Text:** *“Assessment of methodological quality and risk of bias was not performed as synthesis of effect estimates was beyond the scope of this review.”* |
| Summary measures | 13 | State the principal summary measures (e.g., risk ratio, difference in means). | **Page:** Not applicable  **Text:** *This review aimed to map outcome reporting rather than estimate treatment effects; therefore, effect measures were not specified.* |
| Synthesis of results | 14 | Describe the methods of handling data and combining results of studies, if done, including measures of consistency (e.g., I^2^) for each meta-analysis. | **Page: Methods – Data analysis (p.7)**  **Text:** *“Descriptive statistical analysis was performed using Microsoft Excel. The number and proportion of studies reporting each SOT were summarised using frequencies and percentages.”* |

Page 1 of 2

| **Section/topic** | **#** | **Checklist item** | **Reported on page #** |
| --- | --- | --- | --- |
| Risk of bias across studies | 15 | Specify any assessment of risk of bias that may affect the cumulative evidence (e.g., publication bias, selective reporting within studies). | **Page:** Not applicable  **Text:** *Reporting bias assessment was not undertaken as no quantitative synthesis was performed.* |
| Additional analyses | 16 | Describe methods of additional analyses (e.g., sensitivity or subgroup analyses, meta-regression), if done, indicating which were pre-specified. | **Page:** Not applicable |
| **RESULTS** | | |  |
| Study selection | 17 | Give numbers of studies screened, assessed for eligibility, and included in the review, with reasons for exclusions at each stage, ideally with a flow diagram. | **Page:** Results – Included studies (p.8)  **Text:** *“Searches in MEDLINE (via PubMed), Embase, Cumulative index to Nursing and Allied Health Literature (CINAHL), and The Cochrane Library were conducted in June 2025, identifying 8106 records (MEDLINE: 1839, Embase: 4931, CINAHL: 555, Cochrane library: 781). After removing duplicates, 5488 records were screened by title and abstract of which 199 records were identified for retrieval. Full text screening was conducted on 196 articles to determine their eligibility with 59 articles meeting criteria for inclusion. A further 13 articles were identified through citation searching of which ten were included after full text review bringing the total of articles included in the review to 69. A mixed-methods qualitative study identified during the search described the development of an HRQoL tool and its associated outcomes[20]. As this tool was later used in another study[21], the qualitative study was excluded to prevent duplication. The screening process, including reasons for exclusion, is presented in the PRISMA flow diagram* ***(Figure 1)****.”* |
| Study characteristics | 18 | For each study, present characteristics for which data were extracted (e.g., study size, PICOS, follow-up period) and provide the citations. | **Page:** Results – Study characteristics (p.8)  **Text:** *“The 69 articles included in this review represent 67 individual studies. One study reported findings across two articles, another reported HRQoL findings of a subset of participants in a separate article]. Two studies that reported intermediate- and long-term follow-up of participants from earlier studies were retained as separate studies for the purposes of outcome evaluation.*  *Of the 67 included studies, 40 were observational (retrospective or prospective cohort) studies, 23 were interventional studies (of which five were RCTs) and four were systematic reviews. Just eleven studies involved comparisons with other treatment modalities or non-treatment strategies.*  *Across the included studies, 17 different treatment types were identified, the most commonly reported being electrocautery ablation, used in 18 studies. A comprehensive overview of study characteristics for the 67 included studies is provided in* ***Table S2****, with a concise summary presented in* ***Table 2****.”* |
| Risk of bias within studies | 19 | Present data on risk of bias of each study and, if available, any outcome level assessment (see item 12). | **Page:** Not applicable  **Text:** *Risk of bias was not assessed.* |
| Results of individual studies | 20 | For all outcomes considered (benefits or harms), present, for each study: (a) simple summary data for each intervention group (b) effect estimates and confidence intervals, ideally with a forest plot. | **Page:** Results – Outcomes reported (pp.8–10)  **Text:** *“A total of 966 outcomes were reported, grouped into 170 standardised outcome terms across five COMET core areas.”* |
| Synthesis of results | 21 | Present results of each meta-analysis done, including confidence intervals and measures of consistency. | **Page:** Not applicable  **Text:** *This review aimed to map outcome reporting rather than estimate treatment effects; therefore, effect measures were not specified and consequently has no meta-analysis results.* |
| Risk of bias across studies | 22 | Present results of any assessment of risk of bias across studies (see Item 15). | **Page:** Not applicable  **Text:** *Reporting biases were not formally evaluated.* |
| Additional analysis | 23 | Give results of additional analyses, if done (e.g., sensitivity or subgroup analyses, meta-regression [see Item 16]). | **Page:** Not applicable |
| **DISCUSSION** | | |  |
| Summary of evidence | 24 | Summarize the main findings including the strength of evidence for each main outcome; consider their relevance to key groups (e.g., healthcare providers, users, and policy makers). | **Page:** Discussion (pp.11–12)  **Text:** *“Outcome reporting in anal HSIL treatment studies is highly inconsistent, limiting evidence synthesis and comparative analysis.”* |
| Limitations | 25 | Discuss limitations at study and outcome level (e.g., risk of bias), and at review-level (e.g., incomplete retrieval of identified research, reporting bias). | **Page:** Discussion – Limitations (p.12)  **Text:** *“Some limitations must be acknowledged. This study was designed to map the scope and nature of outcome reporting in anal HSIL treatment studies rather than to synthesise effect estimates; accordingly, formal assessment of methodological quality and risk of bias was not undertaken. Such assessments, while essential for meta-analyses, are less informative for a descriptive review of outcome measurement. Despite full concordance during an initial calibration exercise, study selection was undertaken by a single reviewer, and some risk of selection bias cannot be excluded. The review was restricted to full-text articles published in English due to resource limitations and the lack of readily available translation facilities. This may have excluded relevant studies published in other languages, potentially omitting outcome measures used in non-English-speaking settings. However, the 67 included studies represent research from thirteen countries, several of which are non-English-speaking, suggesting that the review captures a degree of global variation in outcome reporting. While some region-specific practices may still be under-represented, the international spread of included studies, together with the diverse expertise within our steering group, should help to minimise any impact on the global applicability of a future COS..”* |
| Conclusions | 26 | Provide a general interpretation of the results in the context of other evidence, and implications for future research. | **Page:** Discussion – Conclusion (p.12)  **Text:** *“In conclusion, this systematic review has identified substantial heterogeneity in outcome reporting in anal HSIL treatment trials and highlights the urgent need for a COS in this disease area. The comprehensive list of outcomes identified in this review is the first step towards the development of this COS, the adoption of which will be instrumental in fostering high-quality, comparable and meaningful research that ultimately improves patient care for individuals affected by anal HSIL.”* |
| **FUNDING** | | |  |
| Funding | 27 | Describe sources of funding for the systematic review and other support (e.g., supply of data); role of funders for the systematic review. | **Page:** Title page (p.2)  **Text:** *“This study forms part of a 3-year PhD project funded by the Department of General Surgery at Lancashire Teaching Hospitals NHS Foundation Trust (year 1), the NIHR Manchester Biomedical Research Centre (BRC) 2022 bid ‘North West’ section (years 2 and 3), and the Rosemere Cancer Charity (years 2 and 3) (Registered Charity Number 1131583). Researcher salary and running expenses are covered by this funding.”* |

*From:*  Moher D, Liberati A, Tetzlaff J, Altman DG, The PRISMA Group (2009). Preferred Reporting Items for Systematic Reviews and Meta-Analyses: The PRISMA Statement. PLoS Med 6(7): e1000097. doi:10.1371/journal.pmed1000097

For more information, visit: **www.prisma-statement.org**.

Page 2 of 2
